# How light reshapes the mind. An active inference framework for the cognitive and emotional effects of indoor lighting

**DOI:** 10.3389/fncom.2026.1865557

**Published:** 2026-08-26

**Authors:** Luca M. Possati

**Affiliations:** Faculty of Behavioural, Management and Social Sciences, University of Twente, Enschede, Netherlands

**Keywords:** active inference, cognition, emotion, free-energy principle, light

## Abstract

Indoor lighting influences cognition, affect, and behavioral regulation, yet these effects are typically studied as isolated empirical findings rather than as components of a unified computational process. This paper proposes an active inference account of the impact of shared indoor lighting—non-personalized illumination in environments occupied by multiple users such as offices, classrooms, and libraries. The central hypothesis is that lighting acts through three computationally distinct channels, each indexed by a metrologically separable descriptor of the light field rather than by correlated color temperature (CCT): an *epistemic* channel driven by task-plane photopic illuminance, an *affective* channel driven by ambient melanopic equivalent daylight illuminance (melanopic EDI; CIE S 026:2018), and a *normative* channel driven by chromaticity. Because melanopic EDI and chromaticity are dissociable by metamerism, and because the affective input is a whole-field, session-integrated quantity whereas the epistemic input is local and instantaneous, the three channels are separable both in computational role and in physical driver. To formalize and test this hypothesis, the paper develops a proof-of-concept partially observable Markov decision process (POMDP) under the active inference framework. The model simulates a cognitive agent performing sustained reading over a 5-h session, receiving observations from two modalities simultaneously: reading performance and eye tracking. Text legibility is a symmetric function of illuminance; the oculomotor signal is asymmetric, degrading rapidly under glare due to pupil constriction, and flat under sub-optimal illuminance. Three factors are crossed in a full factorial design: lighting scenario (warm-dim, moderate, and bright-cool), chronotype (morning, intermediate, and evening), and spatial desk position in a realistically specified room with a simulated photometric field. Six falsifiable predictions are derived from the model structure. These six predictions fall into three tiers by evidential status—structural invariants that verify the implementation (P1, P2, and P6), the critical test of the multi-channel hypothesis (P3, P4), and one exploratory prediction (P5)—and are borne out in simulation at *N*_MC_ = 20 replicates. The central prediction concerns the spatial ordering of performance under intense cool illumination. Under moderate light, performance follows the legibility gradient: the best-lit desk position produces the best output. Under intense cool light, this ordering completely inverts: the desk position with the best legibility in both channels—receiving only 402 lux at the far corner—produces higher reading speed (71.3 wpm) than the position with near-zero legibility in both channels under 1,200 lux of glare (59.9 wpm). The inversion arises from two concurrent mechanisms: the well-lit agent accurately perceives its deteriorating state and rests strategically, while the glare-blinded agent works blindly through its deterioration but pays a direct mechanical speed penalty. In this condition, strategic rest at the far-corner position yields higher total output than blind persistence under severe glare. This prediction requires the multi-channel architecture and is not expected under models in which lighting acts only through arousal or only through monotonic legibility. Additional predictions include a chronotype-specific risk sign-reversal between scenarios, a position-by-chronotype interaction differing by a factor of two between morning and evening types, and a structural invariance of the intermediate chronotype's EFE risk across all scenarios as a direct consequence of setting its affective tolerance factor to zero. The contribution of the paper is theoretical: it does not provide a calibrated model of human reading performance, but a computational framework that links empirical findings on lighting to an explicit generative architecture with testable, non-trivial signatures. In short, the paper offers a computational theory of light as a modulator of inference, arousal, and behavior, and translates it into concrete experimental predictions.

## 1 Introduction

The profound connection between architecture, cognition, emotions, and general wellbeing is now widely recognized and extensively studied (Abbas et al., 2024; Djebbara et al., 2019, 2021; Eberhard, 2009a,b; Pallasmaa, 2015). Recent advances in neuroscience techniques enable a deeper understanding of how architectural design choices impact the human brain and behavior, overcoming previous methodological limitations (Wang et al., 2022). Of all the elements that make up an artificial environment, lighting plays a crucial role (Sholanke et al., 2021; Livingston, 2022; Plummer, 2016). Light is essential for visual and spatial perception and serves as the primary medium for presenting information. Beyond vision, light plays a critical role in regulating circadian rhythms, influencing productivity, shaping social interactions, and enhancing individuals' sense of safety (LeGates et al., 2014; Blume et al., 2019; Dijk and Archer, 2009).

Light exerts significant and well-documented effects on human health, entraining circadian rhythms and modulating sleep, hormonal secretion, mood, and alertness through intrinsically photosensitive retinal ganglion cells, with disruption implicated in circadian misalignment and mood disorders (LeGates et al., 2014; Boyce, 2010, 2022; Blume et al., 2019; Hatori et al., 2017; Kumari et al., 2023; Guarnieri, 2024). These health effects, however, are not our focus; we note them only to situate the cognitive and affective effects that follow.

Light is also a powerful and direct regulator of emotion and cognition. Moderate increases in illuminance reliably enhance alertness, positive affect, and processing speed (Knez, 1995; Shishegar and Boubekri, 2022); cooler, blue-enriched light raises alertness and can improve subjective wellbeing and reaction times (Chellappa et al., 2011; Park et al., 2013; Zhu et al., 2025); and warmer, dimmer light is associated with relaxation and comfort. These effects are not reducible to circadian regulation: Yan et al. (2019) show, in a diurnal rodent, that daytime light intensity regulates mood and cognition through orexin-centered circuits and monoaminergic signaling rather than primarily via circadian disruption. It is these affective and cognitive effects, rather than the broader health consequences of light, that the present paper seeks to explain.

In this study, we examine the effects of shared indoor lighting on human cognition and emotion through the lens of active inference and the Free Energy Principle (FEP) (Parr et al., 2022; Namjoshi, 2026; de Vries, 2026). By *shared lighting*, we mean non-personalized illumination conditions in environments occupied by multiple users, such as open-plan offices, classrooms, and libraries. More specifically, we ask how different shared lighting conditions shape cognition and emotion, and how they modulate the balance between exploration and exploitation, information gain and pragmatic value, and expected ambiguity and risk, as conceptualized in the active inference literature.

The paper's central claim is that active inference provides a useful and comprehensive framework for understanding how different components of light shape behavior. The hypothesis we seek to formalize and test is that light influences human behavior through three distinguishable channels: *an epistemic channel*, mediated by illuminance and affecting the ambiguity or discriminability of perceived objects; *an affective channel*, mediated by correlated color temperature and affecting mood, arousal, or confidence in action; and *a normative channel*, mediated by spectral composition and affecting the action tendencies that are more readily sustained in a given context. We argue that the existing literature supports this hypothesis, although the evidence remains fragmented and largely empirical.

We motivate this partition as follows. The literature associates illuminance most consistently with attentional engagement and the quality of early perceptual processing; spectral and color characteristics most consistently with arousal and affect; and color as such with action-relevant meaning and approach–avoidance tendencies (Elliot, 2015). These three empirical regularities map naturally onto three distinct computational roles in an inferential agent: the precision of perception, the evaluation of outcomes, and the prior over actions. That correspondence, developed formally in Section 3, is the rationale for partitioning lighting into an epistemic, an affective, and a normative channel.

Accordingly, this paper develops a proof-of-concept active inference model designed to establish the conceptual coherence and formal tractability of the three-channel hypothesis, rather than to offer a calibrated or fully predictive account of human behavior. The model is examined in the specific context of reading, using a shared indoor environment and a common cognitive architecture instantiated across three chronotype profiles–lark, intermediate, and owl. The parameter values adopted in the simulations are chosen for qualitative plausibility rather than empirical fit, and the results should be interpreted in that light. Empirical validation therefore remains the necessary next step. A central aim of the present paper is to make that step tractable by specifying what should be measured, under which lighting conditions, and in which participant population.

## 2 Literature review

This narrative review covers empirical studies published between 2000 and January 2026 on the effects of indoor lighting on cognitive and emotional functioning, with particular attention to reading; the search strategy, screening procedure, and quality appraisal are reported in Appendix B. Twenty-five studies met the inclusion criteria and were classified by whether their principal manipulation concerned illuminance, color temperature or spectrum, or their combination.

Research on indoor lighting and cognition has moved through three broad phases. Early work established illuminance thresholds for visual performance and comfort. A second wave, following the identification of melanopsin-containing retinal ganglion cells, established non-visual, spectrally driven effects on alertness and circadian regulation. Recent work has begun to manipulate illuminance, spectrum, and their spatial distribution jointly. We organize the reviewed studies along three dimensions—illuminance, spectral and color quality, and their combination—which structures the synthesis that follows.

Based on this review, several gaps in the literature were identified:

- First, most studies treat light as lux + CCT at a point, ignoring where fixtures are, how many there are, beam spread, wall/ceiling reflectance, and contrast patterns. We almost never know if light is direct/indirect, uniform vs. localized, frontal vs. side-lit, or how daylight and electric light interact—so the results are hard to translate into actual architectural lighting design rather than lab recipes. In other words, there is a lack of architectural sense of lighting, that is, attention to the space in which the light spreads, and in which the light sources are placed.
- Second, lux, CCT, and spectral composition are rarely manipulated and modeled together in a factorial, quantitative way. This means we still do not have a coherent predictive model of how combinations of lux + CCT + spectrum shape attention, memory, and reading; we just have scattered good settings.
- Third, most evidence comes from short sessions (minutes/hours) with young, healthy participants doing simplified tasks (2-back, Go/No-go, and brief reading tests). We know very little about long-term learning, real school performance, or diverse groups (children with reading difficulties, older adults, and neurodivergent learners) in real classrooms or offices over weeks or months. This means we have, at best, a purely laboratory view of these phenomena.
- Fourth, emotions are usually reduced to single self-report scales (comfort, sleepiness). As a result, we lack nuanced understanding of which lux-CCT setups are optimal for which emotional states (calm focus vs. high arousal).

The results that emerge from these studies, therefore, are fragmented (both in terms of content and methodology) and solely empirical (they identify patterns, not mechanisms of causal influence). Taken together, these studies expose a single gap. The literature reliably identifies which lighting settings help which outcomes, but it stops at empirical regularities; it offers no mechanistic account of how a given lighting condition is transformed into perception, evaluation, and action by the occupant. Supplying such an account, and deriving testable signatures from it, is the aim of this paper.

We also conducted a more specific literature review of explanatory models for light effects. The result is that we have only two main explanatory frameworks (they remain implicit in the examined studies or are ignored): (a) non-image-forming (NIF) photoreception via melanopsin-sensitive ipRGCs, which sync circadian rhythms, boost alertness, and curb melatonin, explaining physiological impacts (Vandewalle et al., 2009; Campbell et al., 2023, 2024; Shi et al., 2025; Lasauskaite et al., 2025; Tähkämö et al., 2019); (b) arousal theory (Yerkes–Dodson) according to which light modulates arousal, and thus can improve or impair performance depending on intensity, spectrum, and context (Easterbrook, 1959). Yet, NIF and arousal theory do not fully bridge biology to experience, creating a gap between neural signals and behavior. Light, in this sense, exposes the instability of traditional explanatory hierarchies—it is simultaneously a physical stimulus, a neurochemical regulator, and a phenomenological condition. Treating it exclusively through one framework erases its multilevel nature. A truly integrative model would thus need to conceptualize lighting not as an external variable but as a situated modulation of brain–environment coupling, shaping the organism's readiness for action, affective tone, and cognitive scope.

Now, based on the results of the literature review, a general hypothesis can be put forward: illuminance primarily influences cognitive processes, while Kelvin is more closely associated with emotional responses, albeit in ways that are sensitive to situational context and the type of activity being performed. Spectral composition, in turn, appears to play a normative and modulatory role, shaping how illuminance and Kelvin interact to orient behavior toward particular actions or courses of action in a given context. Cognitive, emotional, and normative dimensions of lighting are not only analytically separable but functionally distinct, and each component can be modeled as exerting a specific and identifiable influence on human experience and behavior.

In the next sections we intend to show how this hypothesis is supported by the literature review, albeit in a purely empirical and fragmented way (see Table 1).

**Table 1:** Overview of the literature review results for each component of light.

Component	Primary effect	Impacts	Decision-related implications
Illuminance (lux)	Modulates attentional engagement and sensory processing	Non-linear, task-dependent effects; moderate levels (≈300–600 lux) support reading, alertness, and processing speed, while very high levels (≈1,000–1,200 lux or more) may impair sustained reading and memory	Indirect influence via alertness and attentional readiness; no direct effects on decision strategy or confidence
Correlated color temperature (Kelvin)	Tunes emotional activation (alertness vs. relaxation) largely independent of lux	Higher Kelvin (≈5,000–6,500) increases alertness and wellbeing; lower Kelvin (≈2,700–3,500) increases comfort and relaxation; limited effects on higher cognition	Indirect modulation of decision confidence through arousal and perceived effort; no direct evidence on confidence judgments
Spectral composition	Regulates physiological readiness and circadian-related arousal	Short-wavelength enrichment reliably enhances vigilance, sustained attention, and response readiness; mixed effects on executive accuracy	Normatively structures decision context, biasing toward fast, action-oriented vs. slow, deliberative decision modes

### 2.1 Illuminance and cognition

Illuminance influences cognition primarily through its effects on attentional engagement, and early sensory processing, rather than by producing uniform improvements across cognitive domains.

In the literature, moderate illuminance levels (approximately 300–600 lux) are most consistently associated with efficient reading performance, stable learning outcomes, sensory processing, and acceptable visual comfort (Zhou and Pan, 2023; Choi and Suk, 2016; Chauca et al., 2024; Li and Yao, 2025; Nolé Fajardo et al., 2023; Kiziltunali, 2023), whereas very high illuminance (around 1,000–1,200 lux or higher) often produces diminishing or even negative effects on sustained reading and memory tasks, plausibly due to glare, visual fatigue, or excessive perceptual stimulation (Naylor and Firth, 2008; Castilla et al., 2023; Huiberts et al., 2015; Ram and Bhardwaj, 2018). In contrast, moderate increases in illuminance reliably enhance alertness, positive affect, and processing speed, and tend to benefit tasks that rely on vigilance, simple response inhibition, or low-to-moderate cognitive load (Zhu et al., 2025; Ru et al., 2021; Shishegar and Boubekri, 2022; Barkmann et al., 2012).

However, these benefits are not robustly observed for working memory, executive control, or long-term memory, where findings are mixed and sometimes reversed (Huiberts et al., 2015; Ru et al., 2021; Zhu et al., 2019). Neurophysiological evidence from EEG and psychophysiological measures shows that higher illuminance alters cortical activation patterns—such as reduced alpha power, changes in frontal theta activity, and modulation of early visual ERP components—indicating shifts in attentional allocation and sensory gain rather than unequivocal cognitive enhancement (Park et al., 2013; Castilla et al., 2023). Importantly, several studies report better memory performance under lower or moderate illuminance than under brighter conditions, suggesting that reduced sensory intensity may support deeper focus by limiting distraction or cognitive interference.

Overall, the literature indicates that illuminance exerts non-linear and task-dependent effects on cognition: it functions as a regulator of arousal and perceptual precision whose cognitive consequences depend critically on task type, difficulty, exposure duration, population, and interaction with other lighting parameters, rather than conforming to a simple monotonic “more light is better” relationship. In the specific case of reading, performance is generally optimized at moderate luminance levels, while very high luminance often yields no additional benefit or slightly impairs reading speed or efficiency. This is coherent with the Yerkes-Dodson curve.^1^

### 2.2 Kelvin, emotions, and confidence in decision making

Across the literature reviewed, correlated color temperature (CCT, expressed in Kelvin)^2^ shows robust and replicable effects on affective state—particularly emotions, alertness, relaxation, and subjective wellbeing—while providing no direct, task-level evidence on confidence in decision making as an explicitly measured outcome (Chellappa et al., 2011; Choi and Suk, 2016; Park et al., 2013; Zhu et al., 2019).

When illuminance is controlled, higher CCT or blue-enriched light (approximately 5,000–6,500 Kelvin) is consistently associated with increased alertness, reduced sleepiness, and, in some studies, improved subjective wellbeing (e.g., joy, contentment, curiosity, and cheerfulness) and visual comfort, effects that are often attributed to short-wavelength sensitivity of non-image-forming photoreceptive pathways. By contrast, lower CCT or warmer light (approximately 2,700–3,500 Kelvin) is more reliably linked to reduced arousal, greater relaxation, and higher perceived comfort (e.g., emotions like calmness, serenity, tranquility, relief, and peacefulness).

Several studies indicate that color temperature modulates arousal independently of illuminance, demonstrating that the spectral quality of light can shape emotional activation even when light intensity is held constant.

With respect to decision-making confidence, the evidence remains indirect: none of the reviewed studies directly measure confidence judgments or metacognitive certainty (Shishegar and Boubekri, 2022). However, because confidence is known to covary with alertness, perceived effort, and emotional activation, the documented CCT-driven shifts in comfort plausibly influence subjective readiness or decisional assurance without guaranteeing improvements in accuracy or executive performance.

Overall, the literature supports the conclusion that Kelvin reliably tunes emotional activation states that may indirectly bias confidence in decision making, while stopping short of demonstrating a direct causal effect on confidence itself.

### 2.3 Spectral composition, chromaticity, and decision context

According to the literature, spectral composition is more directly connected to the normative dimension—that is, in a given context it clarifies which action should be taken (e.g., the green of a traffic light signals when to go). Nevertheless, an overlap with the effects of illuminance and CCT is always possible.

Unlike illuminance or correlated color temperature alone, spectral composition reliably modulates alertness, sleepiness, and arousal even when overall brightness is held constant, with short-wavelength-enriched spectra (blue-cyan range) consistently suppressing melatonin, increasing vigilance, and reducing subjective fatigue across laboratory and field settings (Chellappa et al., 2011; Grant et al., 2021; Zhu et al., 2025; Mott et al., 2012). These effects are robust and replicable, indicating that spectral composition captures biologically meaningful variation that nominal CCT values only approximate.

At the cognitive level, spectral enrichment selectively facilitates sustained attention, response speed, and readiness to act, while effects on higher-order executive accuracy or complex reasoning remain mixed and task-dependent (Chellappa et al., 2011; Keis et al., 2014; Grant et al., 2021).

By regulating baseline arousal, temporal alignment, and perceived effort, spectral composition sets the conditions under which decisions are made, effectively biasing agents toward faster, more action-oriented or vigilance-based decision modes under short-wavelength-rich spectra. Conversely, spectra with reduced short-wavelength content are associated with lower arousal and greater comfort, plausibly favoring slower, more deliberative, or low-pressure decision contexts.

Although the reviewed studies do not explicitly operationalize normativity or decision theory, the consistency of spectral effects on physiological readiness and cognitive availability could support the conclusion that spectral composition functions as a contextual regulator that shapes which kinds of decisions are more likely, more fluent, or more sustainable at a given moment.

This result aligns with Elliot (2015), who shows that color shapes antecedent decision processes–attention, arousal, approach-avoidance motivation, dominance, trust, and risk sensitivity. Red tends to increase avoidance and caution, whereas blue promotes trust, calmness, and alertness. These effects bias framing and readiness rather than decision accuracy, though evidence remains limited.

## 3 Model

### 3.1 Theoretical background: active inference and the free energy principle

This section develops a computational model aimed at testing the hypothesis—namely, the roles of task-plane photopic illuminance, ambient melanopic EDI, and chromaticity in shaping cognition, emotion, and normative behavior.

The model is grounded in the theoretical framework of active inference and the free energy principle (FEP). To keep the analysis tractable, we focus on a specific and representative case: reading in an environment with artificial lighting. Within this scenario, we formalize the three patterns using active inference and derive a set of predictions that should follow *if those patterns hold*. The goal is not merely to redescribe the literature but to show that these three patterns can be unified within a single coherent computational framework, and to make explicit what that framework predicts.

Active inference is a theoretical framework according to which physical random dynamical systems (e.g., living organisms, artifacts, and social systems) maintain their integrity by minimizing surprise, or more precisely a tractable upper bound on surprise known as variational free energy (Friston, 2013, 2019; Parr et al., 2022; Namjoshi, 2026; de Vries, 2026). This framework has been applied especially in the study of the brain (Friston, 2010). Under the FEP, perception, action, and learning are not separate processes but mutually dependent aspects of the same imperative: to keep the system within expected and viable states by continuously reducing uncertainty about the causes of sensory input. In this view, agents do not passively register the world; they actively infer the hidden causes of their sensations and act so as to make their sensory exchanges with the environment more predictable.

Formally, in active inference, an agent is described as possessing a generative model of the world. This model specifies beliefs about hidden states, the observations those states are expected to generate, and the way states evolve over time. Perception corresponds to updating beliefs about hidden states so as to better explain current observations. Action corresponds to selecting policies that are expected to minimize future free energy. Thus, behavior is driven not only by the pursuit of preferred outcomes, but also by the reduction of uncertainty. This is why active inference has two inseparable dimensions: a *pragmatic* dimension, oriented toward valuable or goal-consistent states, and an *epistemic* dimension, oriented toward information gain and uncertainty reduction.

A central quantity in active inference is expected free energy, which evaluates candidate policies in terms of both expected risk and expected ambiguity. Expected risk captures the extent to which a policy is predicted to lead away from preferred outcomes, whereas expected ambiguity captures the uncertainty that is expected to remain under that policy. Policies are therefore favored when they both improve the likelihood of preferred states and reduce uncertainty about the environment. This dual structure is particularly useful for the present study, because reading under different lighting conditions is not only a matter of maximizing performance, but also of modulating perceptual precision, affective stability, and the cost of maintaining effective engagement over time.

The present application of active inference and the FEP is one instance of a broader program, developed in Possati (2026), that uses active inference to model how human beings interact with technology and designed environments.

### 3.2 Methodological caveats and the locus of light

The literature review is the starting point of our model. As in any modeling effort, the first step is to analyse the target system before constructing the model, focusing on the aspects of greatest interest: the position of the light source, the influence of light on reading, and the relationship between the reader's self-monitoring and their actual reading performance. These are idealizations, but idealizations are essential to any model, since they allow us to concentrate on the central features of the target system (Potochnik et al., 2024).

#### 3.2.1 Simplifying assumptions

Our model rests on a set of assumptions appropriate to a proof-of-concept active-inference architecture. Reading is represented as a partially observable process in which the agent's true cognitive state is hidden and discretized into three states, *s*_*t*_ ∈ {focused, distracted, fatigued}. The agent infers this state from two noisy observation streams, performance and eye tracking, whose likelihood precision is modulated by local illuminance. Illuminance therefore affects not only reading output but also the reliability of self-monitoring: *ϕ*_text_ modulates the informativeness of performance observations, while *ϕ*_eye_ modulates that of oculomotor observations. The model also assumes a separate mechanical effect of text legibility on realized reading speed through ℓ_factor_, so that poor legibility can directly slow reading even when the agent chooses to continue. State transitions are Markovian and action-dependent, with time-on-task fatigue and scenario-induced arousal shaping the probability of moving between states. Chronotype modulates preferences over performance outcomes through *C*, while chromaticity biases action tendencies through the policy prior *E*. Finally, the agent does not maximize words per minute directly; it selects policies by minimizing expected free energy, so realized reading speed is an emergent consequence of hidden state, action, lighting-dependent legibility, and inference under uncertainty.

#### 3.2.2 Three precisions, kept apart

A clarification is necessary regarding precision, because in active inference precision is not a single, unitary quantity. Three distinct quantities must be distinguished. The first is *policy precision*, γ, which in the full process-theory formulation (Friston et al., 2017) is inferred dynamically as a function of the agent's internal state and is associated with the discrepancy between policy beliefs before and after evaluating expected free energy. The second is the *precision of the likelihood mapping*—here *ϕ*_text_ and *ϕ*_eye_—which in the canonical formulation can be estimated online from prediction errors (the formal substrate of attention) or learned over slower timescales through the updating of model parameters. The third comprises the *parameters of the matrices themselves* (*A*, *B*, and *D*), which can be learned via Dirichlet priors.

The present model adopts a *known-precision* choice for all three. Policy precision γ(*t, c*) is a closed-form function of scenario-induced arousal *a*_base_ and the chronotype-specific circadian optimum a^*^(*t, c*); it is state-dependent—preserving the modulatory role the process theory assigns to γ—but is not updated iteratively and carries no accumulator analogous to the β term of the full formulation. The likelihood precisions *ϕ*_text_ and *ϕ*_eye_ are computed once, at initialization, as closed-form functions of local illuminance, and the resulting likelihood matrices are held fixed across the trajectory; they are never re-estimated from the agent's own sensory prediction errors. The matrices *A*, *B*, *D* are specified rather than learned, with no Dirichlet updating across trajectories. This is a deliberate modeling decision: it amounts to assuming that the reader possesses an already-acquired perceptual calibration of their sensory conditions as a function of light, and it is consistent with the standard discrete-state formulation of active inference, in which the likelihood mapping is specified rather than inferred and the learning of its precision is introduced as a distinct, optional component.

#### 3.2.3 Where the three channels act

A second clarification concerns the point at which light enters the architecture. In active inference the influence of the environment is, in principle, mediated by observations: the environment generates *o*_*t*_, and the agent updates its beliefs accordingly. In the present model, light acts in part directly on the parameters, and it is worth stating precisely which parameters, and with what legitimacy.

The three channels are not on the same footing, and conflating them overstates the issue. The *affective* channel operates on *C*, the preference vector. Preferences, in any active-inference model, are a component of the generative model and are never themselves objects of inference; modulating them as a function of the chronotypic arousal mismatch (through κ_*c*_) is therefore fully orthodox. The *normative* channel operates on *E*, the policy prior, which is likewise a fixed component of the generative model. Neither channel raises any difficulty. The case requiring transparency is the *epistemic* channel alone: light modulates *ϕ*, and *ϕ* enters the likelihood matrix *A* through which the agent updates its beliefs. Here the epistemic effect of light does not pass through an observation that the agent interprets; it reconfigures the informativeness of the observation channel from outside. We now show that this is not a departure from active inference, but the certainty limit of a fully orthodox hierarchical model.

#### 3.2.4 The epistemic channel as the certainty limit of a hierarchical model

The direct dependence of *ϕ* on illuminance can be derived as the known-precision limit of a two-level generative model in which *ϕ* is *inferred from an observation* rather than supplied exogenously. We specify that model and make the reduction precise; its full instantiation is the natural next step, but the derivation alone establishes the formal legitimacy of the present architecture.

Augment the generative model with a second-level hidden state g∈G encoding the photic quality of the environment—its *glare level*—discretized for concreteness as G={dim,optimal,glare}, $|G|=N_{g}$. The agent does not observe *g* directly; it receives a photic observation $og\in O_{g}$ (a brightness/glare percept) through a column-stochastic second-level likelihood $Ag\in {\left[0,1\right]}^{N_{g}\times N_{g}}$ with prior *Dg*. The two levels share the first-level cognitive state *s*_*t*_, so the joint distribution factorizes hierarchically as


$$\begin{array}{l} P\left(o_{1:T}^{\text{perf}},o_{1:T}^{\text{eye}},og,s_{1:T},g,\pi \right)\\ =\underset{\text{second level}}{\underset{︸}{P\left(og|g\right)P\left(g\right)}}\\ \underset{\text{first level, conditioned on}g}{\underset{︸}{P\left(\pi \right)P\left(s_{1}\right)\overset{T}{\underset{t=1}{\prod }}P\left(o_{t}^{\text{perf}}|s_{t},g\right)P\left(o_{t}^{\text{eye}}|s_{t},g\right)P\left(s_{t}|s_{t-1},\pi_{t}\right)}}. \end{array}$$ (1)

The only modification relative to Equation 5 is that the first-level likelihoods are now conditioned on *g*: each glare level fixes a local illuminance ℓ(*g*) and hence, through the closed-form maps already defined (Equations 6, 7), a precision pair [*ϕ*_text_(ℓ(*g*)), *ϕ*_eye_(ℓ(*g*))].

Inference proceeds in two coupled steps. The agent first updates its belief over the glare level by the same variational rule used at the first level,


$$\begin{array}{l} Q(g)=\mathrm{softmax}\text{​}(\ln A^{g}[o^{g},:]+\ln D^{g}), \end{array}$$ (2)

and then forms the *belief-averaged* likelihood used in the first-level state update. Because the convex combination in Equation 8 is linear in *ϕ*_*m*_, the expectation passes through the matrix construction, so the agent may equivalently average the precision or the matrix:


$$\begin{array}{r} {\bar{\phi }}_{m}=\underset{Q\left(g\right)}{𝔼}\left[\phi_{m}\left(\ell \left(g\right)\right)\right],\\ A_{m}\left({\bar{\phi }}_{m}\right)=\left(1-{\bar{\phi }}_{m}\right)A_{m}^{\text{flat}}+{\bar{\phi }}_{m}A_{m}^{\text{sharp}}\\ =\underset{Q\left(g\right)}{𝔼}\left[A_{m}\left(\phi_{m}\left(\ell \left(g\right)\right)\right)\right],\;m\in \left\{\text{text},\text{eye}\right\}. \end{array}$$ (3)

Since ${\bar{\phi }}_{m}\in \left[0,1\right]$ is a convex average of values in [0, 1], and each *A*_*m*_(·) is a convex combination of two column-stochastic matrices, $A_{m}\left({\bar{\phi }}_{m}\right)$ is itself column-stochastic: the construction is closed under second-level belief averaging and requires no renormalization. In this formulation the epistemic effect of light passes entirely through an observation: light generates *og*, the agent infers *Q*(*g*) via Equation 2, and the informativeness of its first-level channels follows through Equation 3. Nothing acts on the parameters from outside the perception–action loop.

The model used throughout this paper is the certainty limit of this hierarchy, in which the second-level posterior is a point mass on the true glare level. We state the reduction as a proposition.

** Proposition 1** (Known-precision reduction). *Let* g*∈G *denote the glare level fixed by the simulated scenario and desk position, and suppose the second-level posterior is degenerate,* *Q*(*g*) = δ_g, g*_ *(Kronecker delta). Then the belief-averaged likelihoods of Equation 3 coincide exactly with the closed-form likelihoods of the one-level model as shown by Equation 4:*


$$\begin{array}{l} {\bar{\phi }}_{m}=\phi_{m}\left(\ell \left(g*\right)\right),A_{m}\left({\bar{\phi }}_{m}\right)=A_{m}\left(\phi_{m}\left(\ell \left(g*\right)\right)\right),\\ \;m\in \left\{\text{text},\text{eye}\right\}, \end{array}$$ (4)

*and the hierarchical generative model (Equation 1) reduces to the one-level model (Equation 5) with no approximation*.

Proof. Under *Q*(*g*) = δ_g, g*_ the expectation in Equation 3 collapses to a single term, ${\bar{\phi }}_{m}=\underset{g}{\sum }\delta_{g,g*}\phi_{m}\left(\ell \left(g\right)\right)=\phi_{m}\left(\ell \left(g*\right)\right)$, and likewise $A_{m}\left({\bar{\phi }}_{m}\right)=A_{m}\left(\phi_{m}\left(\ell \left(g*\right)\right)\right)$ by the linearity already noted. Substituting a degenerate *Q*(*g*) into Equation 1 makes the second-level factors constant and the conditioning on *g* vacuous, leaving exactly the factors of Equation 5.                                          □

Proposition 1 shows that the known-precision assumption is not a shortcut around active inference but the certainty limit of an orthodox hierarchical model: it is the discrete-state convention that the likelihood mapping is specified rather than learned (Da Costa et al., 2020), with its precision set exogenously as the formal substrate of sensory gain (Feldman and Friston, 2010). The limit is also phenomenologically natural—an experienced reader does not slowly infer whether they sit under glare or in dim light; the calibration is already part of their model, that is, *Q*(*g*) has already concentrated on g*. Two consequences follow. First, the present results require no revision: by Proposition 1 they are recovered exactly when *Q*(*g*) = δ_g, g*_. Second, the obvious endogenization—replacing the degenerate posterior with a genuine *Q*(*g*) updated online via Equation 2—is now a precisely specified extension rather than a vague aspiration, and it coincides with the hierarchical oculomotor extension noted below.

#### 3.2.5 The dynamics: generative process and generative model

A final clarification concerns the transition tensor. The modulation of *B* through arousal and fatigue describes, in the first instance, how the reader's hidden state actually evolves: at this level it is part of the *generative process*, and it is not only legitimate but necessary that light and time-on-task should influence the true dynamics of cognitive state. The same modulated *B* is then also used in the policy rollout that evaluates expected free energy, and so enters the agent's *generative model*; this amounts to assuming that the agent knows its own condition-induced dynamics, consistently with the known-model stance adopted throughout. The implementation makes this explicit: a single transition tensor, rebuilt at each step from the fatigue factor and the arousal level, serves both as the law governing the true state transition and as the predictive model used in planning.

#### 3.2.6 The update equations

It follows that the model contains exactly one iterative update equation in the proper sense: the update of the belief over hidden states, *Q*(*s*_*t*_), which combines the two log-likelihoods with the previous belief and renormalizes through a softmax (Algorithm 1, step 4). This is genuine variational inference and constitutes the perceptual core of the model. In the hierarchical reading above, the second-level update *Q*(*g*) (Equation 2) is an update of identical form, suppressed to its certainty limit (Proposition 1) in the present simulations. Action selection is standard planning-as-inference: expected free energy is evaluated for all policies—accumulating ambiguity from both observation modalities and risk from the performance modality alone—and the action is sampled from the resulting policy distribution weighted by the policy prior *E*. Every other session-varying quantity—γ, *ϕ*_text_, *ϕ*_eye_, the arousal level *a*, the Yerkes–Dodson drift terms, the fatigue factor *f*(*t*), and the mismatch that modulates *C*—is not updated iteratively and not learned, but recomputed from closed-form functions of scenario, position, chronotype, and time. The model therefore performs genuine active inference over the cognitive-state space {focused, distracted, fatigued}, while the three channels through which light acts are an exogenous parametrization of that inference problem—exogenous, by Proposition 1, only in the sense of fixing the certainty limit of an orthodox hierarchy.

Algorithm 1 One simulation step at time *t*. 
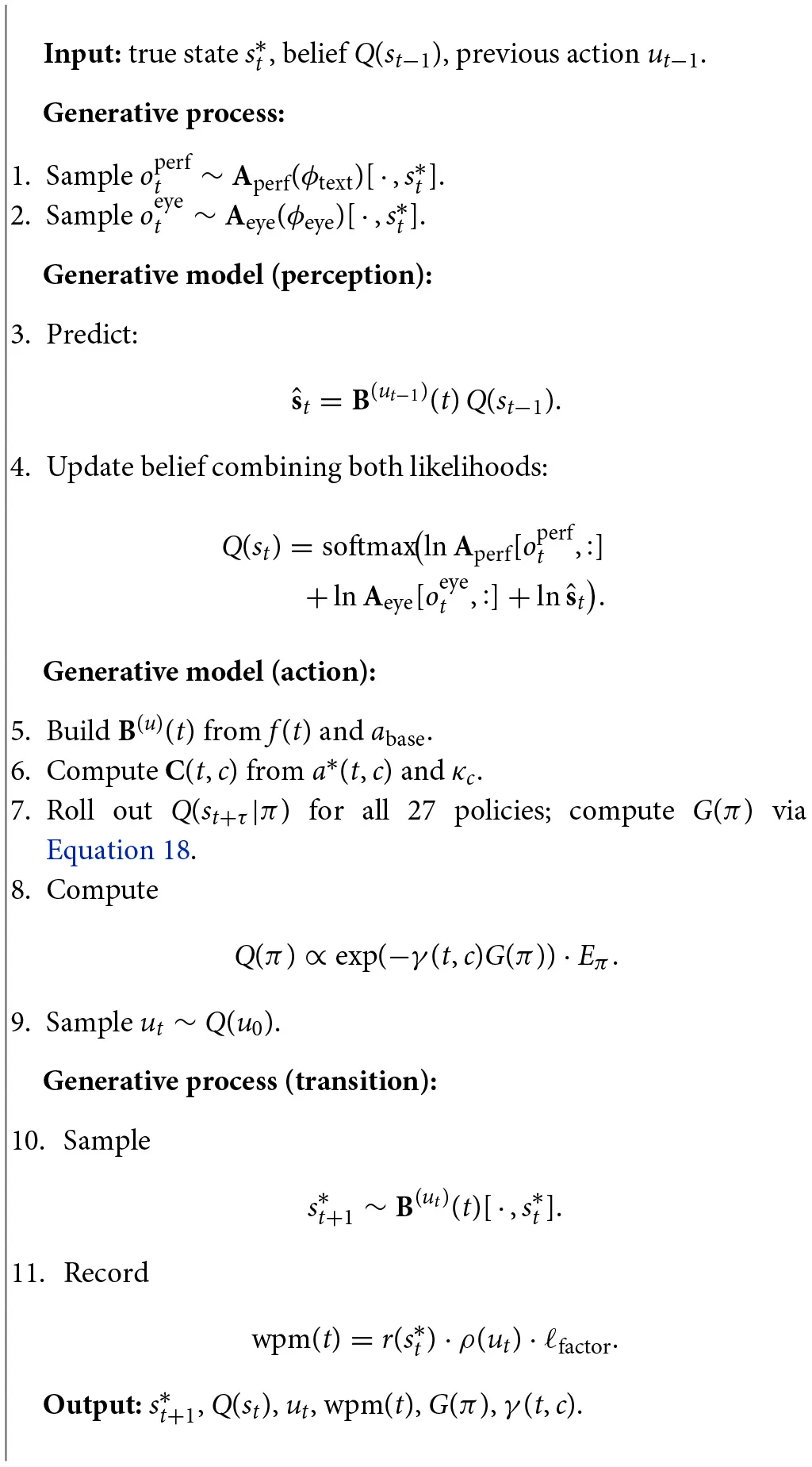

The simulations reveal what follows from these assumptions once they interact over time. They produce model-based knowledge: they show that the three-channel hypothesis is formally implementable, dynamically coherent, and capable of generating non-trivial predictions. They do not yet validate the model against the real system, but they convert a theoretical hypothesis into a set of empirically testable signatures.

### 3.3 Model overview

**Figure 1:**
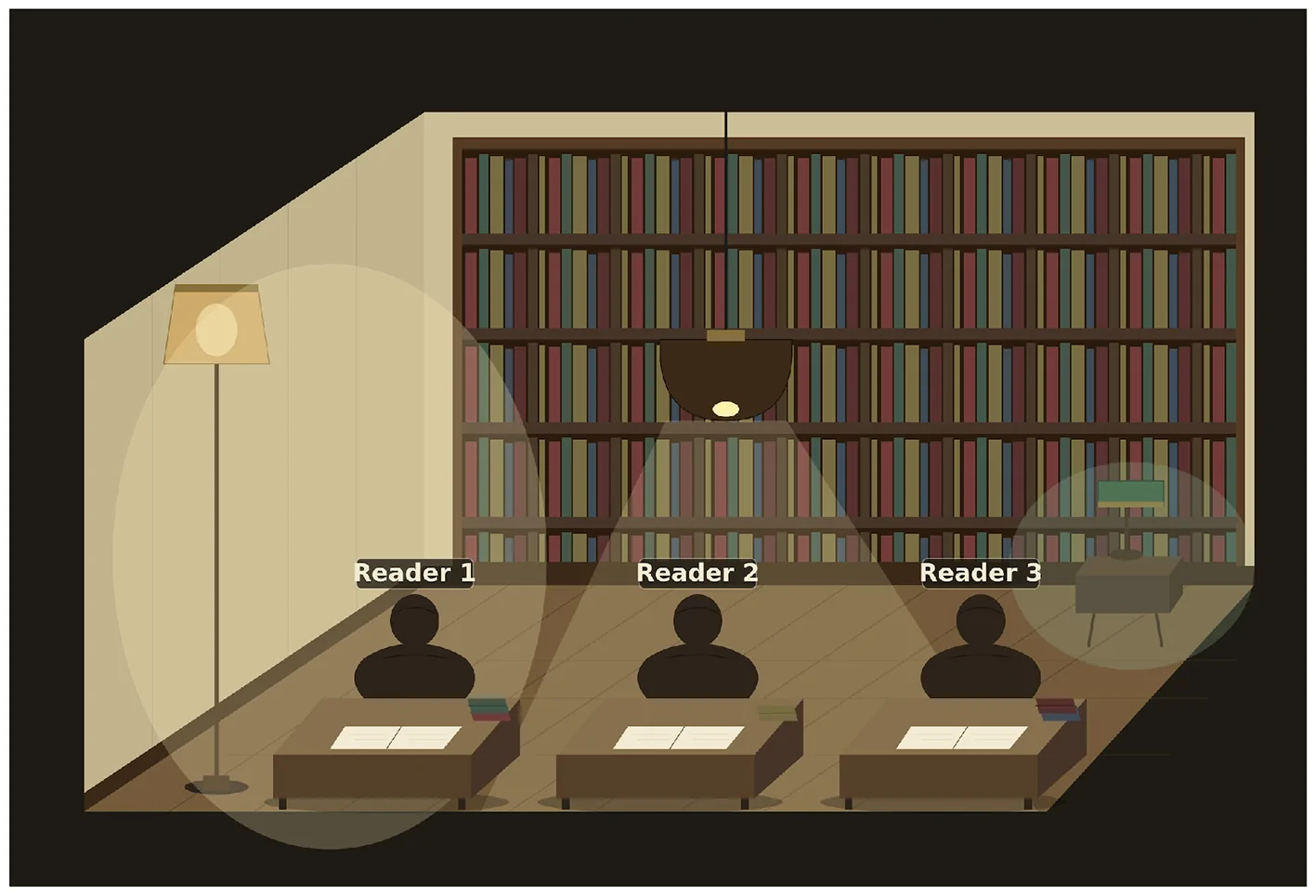
Reading scene with three illumination conditions. Three readers (or three instantiations of the same reader), labeled Reader 1, Reader 2, and Reader 3, are seated at separate tables, each exposed to a distinct light source: a floor lamp adjacent to Reader 1, a ceiling pendant centered above Reader 2, and a small table lamp in the back-right corner near Reader 3.

The model presented here represents a person reading in an office or a library for 5 h. The person cannot directly observe their own cognitive state—they do not know with certainty whether they are focused, distracted, or fatigued. They infer it from two sources simultaneously: how their reading is going (i.e., how fast they are progressing, the fluency of reading), and how their eyes are moving (e.g., whether the eyes are more or less tired). Based on that joint inference, they choose what to do next—keep reading, reread, or take a break. Moreover, in our model, the positions of the light sources and the agent remain fixed; they do not move through space (see Figure 1).

In line with the hypothesis, lighting enters the model in three distinct ways. It affects how informative the person's self-monitoring is: under glare or dim light, observations about one's own performance become noisy and hard to interpret. It affects how comfortable the person is with their current level of activation: a morning-type under bright cool light in the afternoon is over-stimulated relative to their circadian optimum, and this makes them more sensitive to performing poorly. And it nudges behavior directly: warm light creates a mild pull toward resting, blue light toward working.

Over the 5-h session, two things change independently of the lighting. The person's ideal level of activation shifts according to their chronotype—the morning-type's optimum declines through the afternoon while the evening-type's rises. And sustained attention degrades: after 45 mins, the probability of staying focused begins to fall regardless of what the person does.

The agent never optimizes reading speed directly. It minimizes the gap between what it expects to experience and what it prefers to experience. High reading speed is a consequence of being focused and choosing to continue—not a goal the agent pursues.

The model and the simulation formalize this narrative. Their purpose is to derive testable, quantitative predictions from the three-channel hypothesis using a generative model with two observation modalities: performance (reading speed, words per minutes) and eye tracking (oculomotor patterns). The two modalities are affected by lighting differently and provide complementary information to the agent's belief update.

At each decision step (Δ*t* = 20 mins), the agent:

1. Receives two observations simultaneously: $o_{t}^{\text{perf}}$ about its reading performance, and $o_{t}^{\text{eye}}$ about its oculomotor behavior (fixation duration, saccade amplitude, and regression frequency);
2. Updates its belief about its current cognitive state via variational Bayes inference, combining both likelihood signals;
3. Evaluates the Expected Free Energy of all available policies, accumulating ambiguity from both modalities but risk from the performance modality only;
4. Selects an action—continue reading, reread, or pause.

### 3.4 Spaces and variables

Let us move to the detailed description of the model. Table 2 defines all discrete spaces.

**Table 2:** Discrete spaces of the POMDP.

Symbol	Name	Values	Size
*s*_*t*_	Hidden state	focused, distracted, fatigued	*N*_*s*_ = 3
$o_{t}^{\text{perf}}$	Performance observation	high_perf, mid_perf, low_perf	*N*_*o*_ = 3
$o_{t}^{\text{eye}}$	Eye tracking observation	smooth_scan, effortful_scan, degraded_scan	*N*_*e*_ = 3
*u*_*t*_	Action	continue, reread, pause	*N*_*u*_ = 3
π	Policy	sequences $\left(u_{0},u_{1},u_{2}\right)\in U^{3}$	*N*_π_ = 27

#### 3.4.1 Hidden states

Focused: the agent processes new material efficiently (260 wpm). Distracted: the agent reads but frequently loses the thread (150 wpm). Fatigued: sustained-attention capacity is depleted; reading is slow and error-prone (50 wpm). These states are *hidden*—the agent infers them from the two observation streams. The numerical reading rates assigned to the three hidden states should be understood as heuristic state markers rather than empirically fitted estimates. Their role is to establish an ordinal and behaviorally interpretable separation between efficient reading, effortful reading, and severely impaired reading. Thus, the model assumes that a focused agent has high productive capacity, a distracted agent retains partial productive capacity, and a fatigued agent has substantially reduced capacity. The specific values 260, 150, and 50 wpm are chosen to make these state differences explicit in the proof-of-concept simulation, not to provide calibrated estimates of human reading speed.

#### 3.4.2 Performance observations

The performance observation modality provides a coarse-grained signal about the agent's reading fluency. high_perf indicates that reading appears fast, fluent, and stable; mid_perf indicates that reading continues but requires noticeable effort, with reduced fluency or occasional loss of continuity; low_perf indicates slow, disrupted, or re-reading-dominated performance. These categories should not be confused with the realized wpm output of the simulation. They are discrete observations available to the agent for inferring its hidden cognitive state, whereas realized wpm is computed separately from the true state, the selected action, and the lighting-dependent legibility factor.

#### 3.4.3 Eye tracking observations

Smooth_scan: short fixations, wide, regular saccades, no regressions—the oculomotor signature of the focused state. Effortful_scan: longer fixations, narrower saccades, some regressions—consistent with the distracted state. Degraded_scan: many regressions, unstable fixation, tracking errors—the signature of cognitive fatigue. These categories correspond to metrics routinely measured by standard eye trackers: fixation duration, saccade amplitude, and regression count.

#### 3.4.4 Actions

The agent can choose among three actions: *continue, reread*, and *pause*. Each action is associated with a coefficient ρ. Continue: devote the next 20 mins to new material (ρ = 1.0). Reread: review prior material (ρ = 0.5). Pause: take a 20-min break (ρ = 0, allows recovery through **B**). The coefficient ρ is not a probability. It is a productivity multiplier: it determines how much reading output is produced during the current 20-min interval. We introduce ρ to clearly distinguish between the agent's state-dependent reading capacity and the amount of actual forward reading output produced by the selected action.

If the agent chooses *continue*, it reads new material, so all of its current reading capacity counts as forward progress; therefore ρ = 1.0. If the agent chooses *reread*, it reviews material that has already been encountered. This action is still cognitively useful, but it produces less forward progress through the text; therefore the model counts it as half-productive, with ρ = 0.5. If the agent chooses *pause*, it does not read during that interval, so immediate reading output is zero and ρ = 0.

The effect of ρ appears later in the output equation:


$$wpm\left(t\right)=r\left(s_{t}^{*}\right)\rho \left(u_{t}\right)\ell_{factor}.$$

Here, ρ(*u*_*t*_) scales the reading output according to the selected action. Thus, ρ controls immediate productivity, while the transition tensor *B* controls how the action affects the next hidden cognitive state. For example, pausing produces no words per minute in the present step, but it may increase the probability of recovery in the next step through *B*.

Table 3 summarizes the functional role of the main components. The generative model specifies the agent's internal probabilistic architecture, whereas the generative process specifies the simulated world in which a true state $s_{t}^{*}$ generates observations, evolves under action, and produces realized reading output. This distinction is essential because the agent acts on beliefs *Q*(*s*_*t*_), while actual performance depends on the true state $s_{t}^{*}$.

**Table 3:** Core components of the model and their functional role.

Component	Level	Function	Why it is needed
*s*_*t*_ / $s_{t}^{*}$	Hidden state/true state	Represents whether the agent is focused, distracted, or fatigued.	Separates the agent's inferred state from its actual cognitive condition.
$o_{t}^{perf}$, $o_{t}^{eye}$	Observations	Provide performance and eye-tracking evidence about the hidden state.	Allow the agent to infer its cognitive state indirectly, rather than observing it directly.
*A*^*perf*^, *A*^*eye*^	Likelihoods	Encode *P*(*o*^*perf*^∣*s*) and *P*(*o*^*eye*^∣*s*). Their precision is modulated by lighting.	Implement the epistemic channel: lighting changes how informative self-monitoring is.
ϕ_*text*_, *ϕ*_*eye*_	Precision factors	Control whether the likelihoods are sharp and informative or flat and ambiguous.	Formalize the effect of illuminance on perceptual precision and expected ambiguity.
*B*^(*u*)^(*t*)	Transition tensor	Encodes *P*(*s*_*t*+1_∣*s*_*t*_, *u*_*t*_), the action-dependent evolution of cognitive state.	Allows fatigue, recovery, distraction, and arousal-driven state changes to be modeled.
*f*(*t*)	Fatigue modulation	Shifts *B*^(*u*)^(*t*) from fresh to tired dynamics over the session.	Introduces time-on-task fatigue independently of lighting.
δ_under_(*a*), δ_over_(*a*)	Arousal modulation	Destabilize focus under too little or too much arousal.	Implements the Yerkes–Dodson assumption within the transition dynamics.
*C*(*t, c*)	Preferences	Encodes chronotype-dependent log-preferences over performance outcomes.	Implements the affective channel: arousal mismatch changes how low performance is evaluated.
*D*	Initial prior	Specifies the initial belief over hidden states.	Provides the starting point for inference.
*E*_π_	Policy prior	Biases policies toward continue, reread, or pause depending on spectral context.	Implements the normative channel: lighting biases action tendencies.
*G*(π), *Q*(π)	Policy evaluation	Expected free energy evaluates policies; *Q*(π) turns this evaluation into policy beliefs.	Connects inference to action selection through risk, ambiguity, and prior action bias.
ρ(*u*_*t*_)	Action productivity	Converts actions into immediate reading output: continue = 1, reread = 0.5, pause = 0.	Distinguishes actions that have different productive consequences.
ℓ_*factor*_	Mechanical output factor	Converts text legibility into a direct speed penalty on realized reading.	Separates epistemic uncertainty from the physical effect of poor legibility on wpm.
*wpm*(*t*)	Behavioral output	Records realized reading speed as $r\left(s_{t}^{*}\right)\rho \left(u_{t}\right)\ell_{factor}$.	Provides the observable performance outcome without making wpm the agent's explicit objective.

### 3.5 Generative model

**Figure 2:**
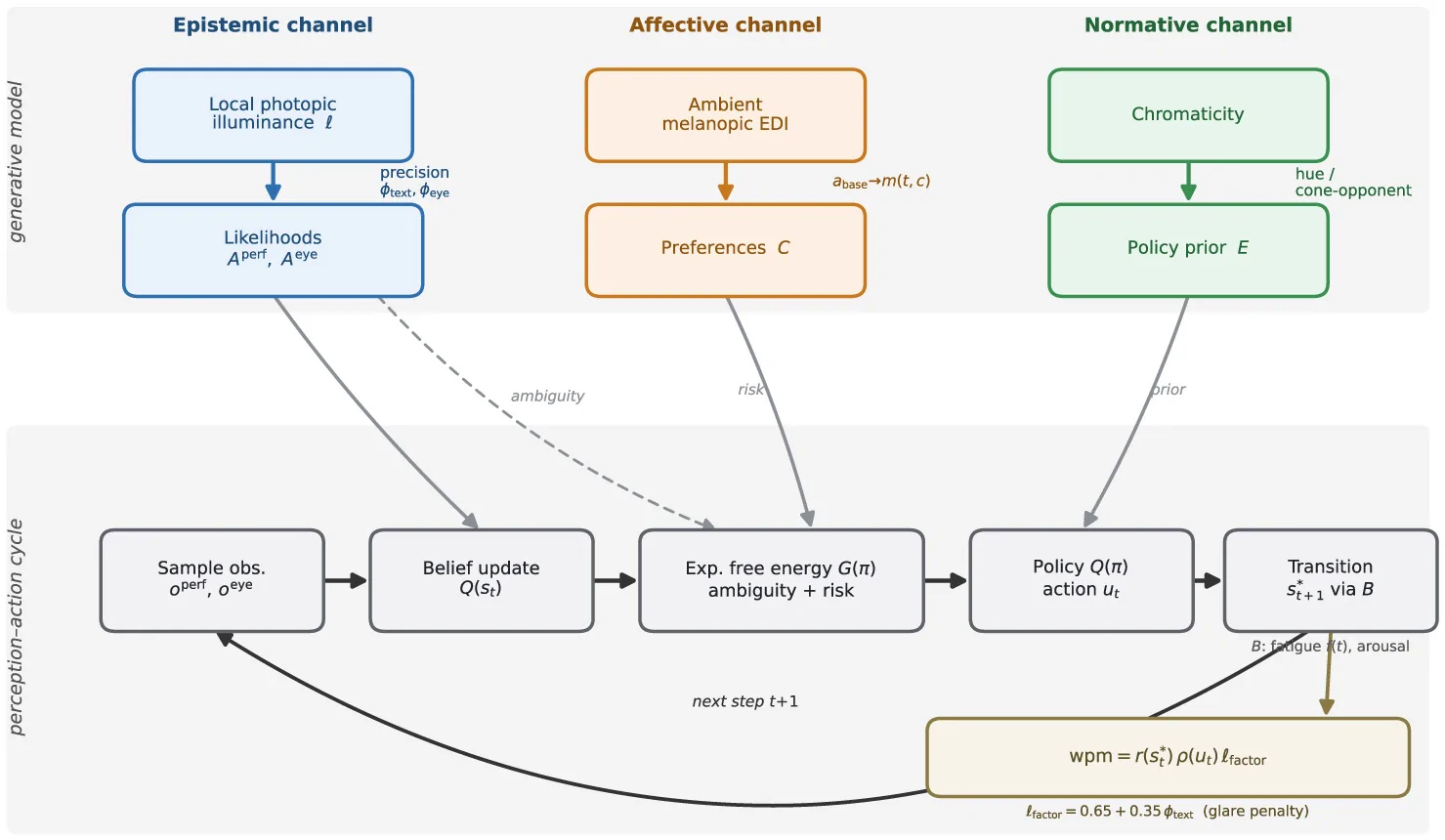
Schematic of the generative model and one simulation step. Light enters at three points: local (task-plane) photopic illuminance sets the precision *ϕ*_text_, *ϕ*_eye_ and hence the likelihood matrices *A*^perf^, *A*^eye^ (epistemic channel); ambient melanopic EDI sets the scenario arousal *a*_base_ and, via the mismatch *m*(*t, c*), the preference vector *C* (affective channel); and chromaticity sets the policy prior *E* (normative channel). The two observation modalities feed the belief update *Q*(*s*_*t*_); expected free energy is evaluated over policies, accumulating ambiguity from both modalities and risk from performance only; an action is sampled; and the true state transitions through *B*, which is itself modulated by fatigue *f*(*t*) and arousal.

The generative model is a joint distribution over both observation streams, hidden states, and policies (here we follow Parr et al., 2022; for an overview, see Figure 2):


$$\begin{array}{r} P\left({\text{o}}_{1:T}^{\text{perf}},{\text{o}}_{1:T}^{\text{eye}},{\text{s}}_{1:T},\pi \right)\\ =P\left(\pi \right)P\left(s_{1}\right)\overset{T}{\underset{t=1}{\prod }}P\left(o_{t}^{\text{perf}}|s_{t}\right)P\left(o_{t}^{\text{eye}}|s_{t}\right)P\left(s_{t}|s_{t-1},\pi_{t}\right). \end{array}$$ (5)

The six factors correspond to (**A**_perf_, **A**_eye_, **B**, **C**, **D**, **E**), described below.

#### 3.5.1 Likelihood matrices **A**_perf_ and **A**_eye_—epistemic channel

Both likelihood matrices are column-stochastic and modulated by light-dependent precision factors. They are the joint entry point of the epistemic channel.^3^

##### 3.5.1.1 Performance likelihood **A**_perf_

Modulated by the text legibility precision factor *ϕ*_text_(ℓ), a symmetric Gaussian centered at the optimal illuminance:


$$\begin{array}{l} \phi_{\text{text}}\left(\ell \right)=\exp\left(-\frac{{\left(\ell -\ell^{*}\right)}^{2}}{\tau_{\ell }^{2}}\right),\;\ell^{*}=500\;\text{lux},\tau_{\ell }=400\;\text{lux}. \end{array}$$ (6)

Both insufficient illuminance and glare degrade text legibility symmetrically. At *ϕ*_text_ = 1 (S2/center), the agent has clear access to its reading performance. At *ϕ*_text_ = 0.047 (S3/center, 1,200 lux), observations are nearly uninformative.

The factor ℓ_*factor*_ should be distinguished from the likelihood precision parameters. Whereas *ϕ*_*text*_ modulates the informativeness of the performance observation channel through *A*^*perf*^, ℓ_*factor*_ captures the direct mechanical effect of text legibility on realized reading speed. In other words, poor lighting does not only make performance observations less reliable; it can also slow reading itself by reducing contrast, increasing line-tracking errors, and inducing glare-related regressions. For this reason, realized output is computed as


$$wpm\left(t\right)=r\left(s_{t}^{*}\right)\rho \left(u_{t}\right)\ell_{factor},$$

where ℓ_*factor*_ = 0.65+0.35ϕ_*text*_ imposes a bounded speed penalty. At optimal legibility, *ϕ*_*text*_ = 1 and ℓ_*factor*_ = 1, so no mechanical penalty is applied. When legibility is very poor, ℓ_*factor*_ approaches 0.65, meaning that reading speed is reduced but not eliminated. Thus, *ϕ*_*text*_ has an epistemic role in the likelihood matrix, while ℓ_*factor*_ has a mechanical role in the production of actual reading output. (More on this later)

##### 3.5.1.2 Eye tracking likelihood **A**_eye_

Modulated by a distinct, asymmetric precision factor *ϕ*_eye_(ℓ):


$$\begin{array}{l} \phi_{\text{eye}}\left(\ell \right)=\{\begin{array}{ll} \phi_{\text{floor}} & \ell \leq \ell^{*}\\ \phi_{\text{floor}}\exp\left(-\frac{{\left(\ell -\ell^{*}\right)}^{2}}{\tau_{\text{eye}}^{2}}\right) & \ell >\ell^{*} \end{array}, \end{array}$$ (7)

with *ϕ*_floor_ = 0.40 and τ_eye_ = 300 lux. *ϕ*_floor_ is the parameter that sets the baseline precision of the eye-tracking channel when illuminance is at or below the optimal level.

Mathematically, however, *ϕ*_floor_ is not a true global floor. It is better understood as a baseline or plateau precision parameter:


$$\phi_{\text{eye}}^{\text{base}}=0.40$$

or equivalently:


$$\phi_{\text{eye}}^{\text{plateau}}=0.40.$$

The asymmetry is physiologically motivated. Saccade metrics and fixation durations are largely unaffected by under-illumination—the oculomotor system is robust to dim conditions and continues to produce cognitively informative patterns. However, pupil constriction under glare progressively masks the cognitive dilation signal above ≈800 lux: at 843 lux (S3/near_source), *ϕ*_eye_ = 0.108; at 1200 lux (S3/center), *ϕ*_eye_ = 0.002, effectively eliminating the oculomotor signal. Below 500 lux, *ϕ*_eye_ is constant at 0.40.

##### 3.5.1.3 Modulation

Both matrices are constructed as convex combinations of flat (uninformative) and sharp (discriminative) base matrices:


$$\begin{array}{l} {\text{A}}_{m}\left(\phi_{m}\right)=\left(1-\phi_{m}\right){\text{A}}_{m}^{\text{flat}}+\phi_{m}{\text{A}}_{m}^{\text{sharp}},\;m\in \left\{\text{perf},\text{eye}\right\}, \end{array}$$ (8)

where


$$\begin{array}{l} {\text{A}}_{\text{perf}}^{\text{sharp}}=\left(\begin{array}{ccc} 0.80 & 0.10 & 0.05\\ 0.15 & 0.70 & 0.20\\ 0.05 & 0.20 & 0.75 \end{array}\right),\;{\text{A}}_{\text{eye}}^{\text{sharp}}=\left(\begin{array}{ccc} 0.75 & 0.15 & 0.05\\ 0.20 & 0.65 & 0.25\\ 0.05 & 0.20 & 0.70 \end{array}\right). \end{array}$$ (9)

(Rows: observations; columns: states focused/distracted/fatigued, see Equation 9).

The uninformative base matrices have uniform columns,


$$A_{m}^{\text{flat}}=\left(\begin{array}{ccc} 1/3 & 1/3 & 1/3\\ 1/3 & 1/3 & 1/3\\ 1/3 & 1/3 & 1/3 \end{array}\right),\;m\in \left\{\text{perf},\text{eye}\right\},$$

so that at *ϕ*_*m*_ = 0 observations carry no information about the hidden state. As a worked example, at *ϕ*_text_ = 0.5 the performance likelihood is the entrywise average of $A_{\text{perf}}^{\text{sharp}}$ and $A_{\text{perf}}^{\text{flat}}$,


$$A^{\text{perf}}\left(0.5\right)\approx \left(\begin{array}{ccc} 0.57 & 0.22 & 0.19\\ 0.24 & 0.52 & 0.27\\ 0.19 & 0.27 & 0.54 \end{array}\right),$$

which remains column-stochastic. In every likelihood matrix, rows index observations and columns index the hidden states (focused, distracted, and fatigued); the same convention applies to *A*^eye^.

Equation 8 should be read as a precision-weighted interpolation between two limiting cases. The matrix $A_{m}^{flat}$ represents an uninformative observation model: observations are only weakly diagnostic of the hidden state, so the agent receives little evidence about whether it is focused, distracted, or fatigued. By contrast, $A_{m}^{sharp}$ represents an informative observation model: observations are strongly aligned with the hidden state, so focused states tend to generate high-performance or smooth-scanning observations, while fatigued states tend to generate low-performance or degraded-scanning observations. The precision parameter *ϕ*_*m*_ ∈ [0, 1] determines how much weight is assigned to each case. When *ϕ*_*m*_ = 0, the likelihood reduces to the flat matrix, $A_{m}=A_{m}^{flat}$, and the observation channel is maximally ambiguous. When *ϕ*_*m*_ = 1, the likelihood reduces to the sharp matrix, $A_{m}=A_{m}^{sharp}$, and the observation channel is maximally discriminative. Intermediate values of *ϕ*_*m*_ produce intermediate likelihoods. Thus, lighting modulates not the hidden state directly, but the informativeness of the observations through which the agent infers that state.

Two points of construction deserve comment, since they are sometimes queried. First, the two observation modalities are kept as separate likelihood matrices, *A*^perf^ and *A*^eye^, exactly as in standard discrete active inference; the interpolation operates *within* each modality, not in place of having two. Second, we use a precision-weighted interpolation between a fixed uninformative matrix and a fixed informative matrix, rather than hand-coding a distinct matrix for each lighting level, because it lets a single continuous physical variable—local illuminance—modulate the informativeness of the observation model continuously. This is the discrete-state analog of sensory precision, the formal substrate of attention (Feldman and Friston, 2010; Da Costa et al., 2020): light does not change the observation model's identity, only its precision. A bank of separately specified matrices would reproduce this at a discrete set of points while obscuring the single degree of freedom that light controls. The transition tensor *B* is built on the same principle (Equation 11): it interpolates between specified fresh and tired tensors as a function of time-on-task, with an additional arousal-dependent drift, so that fatigue and arousal enter the dynamics as continuous, interpretable modulations. The key property of the two-modality design is that high illuminance does not have a uniform epistemic effect across space. Under S3, the center position receives severe glare, which suppresses both the performance and eye-tracking channels, whereas the far-corner position preserves high text legibility and a moderately informative oculomotor signal. Both modalities are compromised by glare, leaving the agent essentially blind to its own cognitive state. At S3/far_corner: *ϕ*_text_ = 0.941 and *ϕ*_eye_ = 0.400—the agent retains good text legibility and moderate oculomotor signal, allowing accurate self-monitoring.

Both modalities contribute to the *ambiguity* term of the EFE (see Equation 10):


$$\begin{array}{l} \text{Ambiguity}\left(\tau \right)={\text{𝔼}}_{Q\left(s|\pi \right)}\left[H\left[P\left(o^{\text{perf}}|s\right)\right]\right]+{\text{𝔼}}_{Q\left(s|\pi \right)}\left[H\left[P\left(o^{\text{eye}}|s\right)\right]\right] \end{array}$$ (10)

Only the performance modality contributes to the *risk* term, because agents do not have explicit preferences over oculomotor patterns—they prefer good performance outcomes, not particular fixation durations.

In other words, the two modalities play different roles in the EFE decomposition. Both performance and eye-tracking observations contribute to expected ambiguity, because both determine how informative future observations are expected to be about hidden cognitive states. However, only the performance modality contributes to expected risk. As mentioned, this is because the agent has preferences over reading-performance outcomes, but no intrinsic preference for particular oculomotor patterns. Eye movements are therefore treated as epistemic evidence about hidden state, not as pragmatic outcomes to be pursued.

#### 3.5.2 Transition tensor **B**

${\text{B}}^{\left(u\right)}\left(t\right)\in {\left[0,1\right]}^{N_{s}\times N_{s}}$ encodes *P*(*s*_*t*+1_∣*s*_*t*_, *u*) and is time-varying through two independent mechanisms as shown by Equations 11, 12. Two fundamental elements here (for an overview of the loop, see Figure 2):

##### 3.5.2.1 Fatigue modulation


$$\begin{array}{l} {\text{B}}^{\left(u\right)}\left(t\right)=f\left(t\right){\text{B}}_{\text{fresh}}^{\left(u\right)}+\left(1-f\left(t\right)\right){\text{B}}_{\text{tired}}^{\left(u\right)}, \end{array}$$ (11)

where the fatigue factor is piecewise:


$$\begin{array}{l} f(t)=\{\begin{array}{ll} 1 & t_{\text{min}}<45\\ \frac{1}{1+\exp(6(\tilde{t}-0.5))} & \text{otherwise} \end{array},\;\tilde{t}=\frac{t_{\text{min}}-45}{255} \end{array}$$ (12)

The fatigue factor *f*(*t*) should be interpreted as a session-level modulation of the transition dynamics—the value indicates how close the system is to the “fresh” state rather than the “tired” state. For the first 45 mins of the session, *f*(*t*) = 1, so the transition tensor remains fully governed by the “fresh” dynamics $B_{fresh}^{\left(u\right)}$. After 45 mins, *f*(*t*) decreases gradually according to a sigmoid function, thereby increasing the relative weight of the “tired” dynamics $B_{tired}^{\left(u\right)}$. This does not mean that the agent necessarily becomes fatigued after 45 mins. Rather, it means that, as time-on-task increases, the transition probabilities increasingly favor deterioration from focused states toward distracted or fatigued states.

##### 3.5.2.2 Yerkes–Dodson arousal modulation

As shown by Equations 13, 14, the focused column of ${\text{B}}_{\text{fresh}}^{\left(u\right)}$ is further modified by the scenario-induced arousal *a*:


$$\begin{array}{ll} \delta_{\text{under}}\left(a\right) & =\mathrm{clip}\left(1.5\max\left(0,0.55-a\right),0,0.35\right), \end{array}$$ (13)

$$\begin{array}{ll} \delta_{\text{over}}\left(a\right) & =\mathrm{clip}\left(2.0\max\left(0,a-0.78\right),0,0.35\right). \end{array}$$ (14)

Scenario S2 (*a* ≈ 0.78) is the neutral point where both drift terms are zero. S1 (*a* ≈ 0.32) induces distraction drift; S3 (*a* ≈ 1.00) induces fatigue drift.

In other words, the Yerkes–Dodson modulation introduces an arousal-dependent drift into the transition tensor *B*. Its purpose is to encode the assumption, justified by the literature, that focus is most stable at an intermediate level of arousal, whereas both under-arousal and over-arousal make focus less stable. When arousal is too low, δ_under_(*a*) > 0, increasing the tendency to drift from focus toward distraction. When arousal is too high, δ_over_(*a*)>0, increasing the tendency to drift from focus toward fatigue. At the neutral arousal level, both drift terms are zero, so no additional arousal-related pressure is added to the transition dynamics. Thus, this mechanism affects how the hidden cognitive state evolves over time, not how observations are generated. Again, also in this case the literature motivates the shape and direction of the mechanism, but not the exact numerical values. The structure is literature-guided; the numbers are heuristic/proof-of-concept.

Another remark is important. The arousal parameter *a* should not be understood as a direct function of local task-plane illuminance. Consistent with the affective channel's descriptor, it is a scenario-level variable summarizing the non-image-forming activation induced by the ambient melanopic EDI of the environment—the whole-field, eye-level melanopic stimulus to which the occupant is adapted—rather than by the peak horizontal illuminance at any one desk. This variable affects the transition tensor *B* by changing the stability of the focused state: under-arousing scenarios (low ambient melanopic EDI) increase drift toward distraction, whereas over-arousing scenarios (high ambient melanopic EDI) increase drift toward fatigue. Thus, *B* captures the dynamic effect of lighting-induced arousal on hidden-state transitions, while *C*—as we will see—captures how chronotype-specific arousal mismatch changes the agent's preferences over performance outcomes.

#### 3.5.3 Log-preference vector **C**—affective channel

The vector *C*(*t, c*) encodes the agent's prior preferences over performance observations and constitutes the affective channel of the model. Whereas the likelihood matrices *A*^*perf*^ and *A*^*eye*^ determine how informative observations are, *C* determines how desirable different performance outcomes are expected to be. In the present model, these preferences are allowed to vary with time and chronotype because the affective cost of poor performance depends on the mismatch between the arousal induced by the lighting scenario and the agent's chronotype-specific arousal optimum. Thus, *C* does not change what the agent observes; rather, it changes how strongly the agent evaluates low-performance outcomes as costly under different lighting and chronotype conditions. The mismatch between light arousal and chronotype changes mainly the tolerance to low performance, not the desire to perform well. The core idea is that all agents prefer high performance. This is fairly stable. The difference between lark, intermediate, and owl emerges instead when things start to go wrong.

$\text{C}\left(t,c\right)\in {\mathbb{R}}^{N_{o}}$ encodes prior preferences over performance observations as log probabilities. **C** is the entry point of the affective channel.^4^ It is time-varying, modulated by the signed arousal mismatch $m\left(t,c\right)=a_{\text{base}}-a^{*}\left(t,c\right)$ as shown by Equations 15, 16:


$$\begin{array}{l} C_{\text{low}}\left(t,c\right)=C_{\text{low}}^{\text{base}}\left(c\right)+\kappa_{c}\cdot m\left(t,c\right). \end{array}$$ (15)

The tolerance factors κ_*c*_ encode how each chronotype evaluates performance under non-optimal arousal:


$$\begin{array}{l} \kappa_{\text{lark}}=-3.0,\;\kappa_{\text{int.}}=0.0,\;\kappa_{\text{owl}}=+3.5. \end{array}$$ (16)

The intermediate chronotype has κ = 0: its preferences are not modulated by the arousal mismatch, making its risk invariant across scenarios.

The tolerance factors κ_*c*_ are treated here as chronotype-specific constants. This is a proof-of-concept simplification: κ_*c*_ could be made state-dependent—for instance, increasing in magnitude with cumulative time-on-task or with the running fatigue factor *f*(*t*), so that arousal mismatch becomes more aversive as stress accumulates—yielding a non-linear affective response. The sensitivity analysis (Appendix C) indicates that the structural predictions depend on the sign of κ_*c*_ rather than on its exact magnitude, so such a refinement would sharpen quantitative comfort predictions without disturbing the qualitative signatures.

In other words, the parameter κ_*c*_ determines how strongly, and in which direction, the arousal mismatch *m*(*t, c*) changes the agent's tolerance for low-performance outcomes. Positive values of κ_*c*_ make low performance more tolerable when lighting-induced arousal exceeds the chronotype-specific optimum, whereas negative values make low performance more aversive under the same mismatch.

Because *C*(*t, c*) is recomputed at each step from *a*_base_ and a^*^(*t, c*), the affective channel is intrinsically time-varying through the circadian optimum, and it accommodates dynamic (tunable) lighting without any architectural change: substituting a time-varying setting *a*_base_(*t*) for the fixed scenario value suffices to model Human-Centric Lighting schedules in which spectral and intensity parameters shift over the session (Čupkovǎ et al., 2019).

#### 3.5.4 Initial prior **D** and policy prior **E**—normative channel

**D** = (0.70, 0.20, 0.10)^⊤^ is fixed across all conditions. The policy prior **E** is the entry point of the normative channel. The context label σ ∈ {warm, neutral, *and*blue} is a coarse partition of chromaticity (the cone-opponent color signal), not of melanopic content; this is what keeps the normative channel dissociable from the affective one, as shown by Equation 17:


$$\begin{array}{l} p_{\text{act}}\left(\sigma \right)=\{\begin{array}{ll} \left(0.30,0.30,0.40\right) & \sigma =\text{warm (pause-biased)}\\ \left(1/3,1/3,1/3\right) & \sigma =\text{neutral}\\ \left(0.45,0.35,0.20\right) & \sigma =\text{blue (continue-biased)} \end{array}. \end{array}$$ (17)

#### 3.5.5 Expected free energy and action selection

For policy π = (*u*_0_, *u*_1_, *u*_2_):


$$\begin{array}{l} G\left(\pi \right)=\overset{H}{\underset{\tau =1}{\sum }}\underset{\text{total ambiguity (both modalities)}}{\underset{︸}{{𝔼}_{Q\left(s_{t+\tau }|\pi \right)}\left[H\left[P\left(o^{\text{perf}}|s\right)\right]+H\left[P\left(o^{\text{eye}}|s\right)\right]\right]}}+\\ \;\underset{\text{risk (performance only)}}{\underset{︸}{D_{\text{KL}}\left[Q\left(o_{t+\tau }^{\text{perf}}|\pi \right)||P^{*}\left(o^{\text{perf}}\right)\right]}}. \end{array}$$ (18)

Policy selection and action selection follow the standard active inference formulation as expressed by Equations 18–20:


$$\begin{array}{r} Q\left(\pi \right)\propto \exp\left(-\gamma \left(t,c\right)G\left(\pi \right)\right)\cdot E_{\pi },\\ \gamma \left(t,c\right)=\gamma_{0}+\gamma_{1}\exp\left(-\frac{{\left(a_{\text{base}}-a^{*}\left(t,c\right)\right)}^{2}}{\tau_{a}^{2}}\right). \end{array}$$ (19)

Instantaneous reading output is computed as


$$\begin{array}{l} \text{wpm}\left(t\right)=r\left(s_{t}^{*}\right)\rho \left(u_{t}\right)\ell_{\text{factor}},\;\ell_{\text{factor}}=0.65+0.35\phi_{\text{text}}. \end{array}$$ (20)

Here, $s_{t}^{*}$ denotes the true hidden cognitive state at time *t*, and $r\left(s_{t}^{*}\right)$ gives the state-dependent baseline reading rate:


r=(260,150,50) wpm

for focused, distracted, and fatigued states, respectively. The term ρ(*u*_*t*_) is the productivity multiplier associated with the selected action:


ρ=(1.0,0.5,0.0),

corresponding to continue, reread, and pause. Thus, cognitive state determines the potential reading rate, while action determines how much of that potential is expressed as productive reading output.

The factor ℓ_factor_ introduces a separate visual-legibility penalty. This term captures the fact that reading speed can be reduced by poor visual conditions even when the agent is cognitively focused and selects a productive action. In this sense, the model distinguishes cognitive capacity from mechanical reading efficiency. Whereas *ϕ*_text_ modulates the informativeness of performance observations in the likelihood matrix *A*^*perf*^, ℓ_factor_ uses the same text-legibility variable to impose a direct penalty on realized reading speed. This penalty represents visual-mechanical effects such as glare, reduced contrast, unstable line tracking, and increased regressions.

The mapping:


$$\ell_{\text{factor}}=0.65+0.35\phi_{\text{text}}$$

bounds the legibility factor between 0.65 and 1.0. When *ϕ*_text_ = 1, text legibility is optimal and ℓ_factor_ = 1, so no mechanical penalty is applied. When *ϕ*_text_ = 0, reading speed is reduced to 65% of its state- and action-dependent baseline, but it is not eliminated. For example, at *S*3/center, where *ϕ*_text_ = 0.047, the legibility factor is:


$$\ell_{\text{factor}}=0.65+0.35\left(0.047\right)=0.666,$$

meaning that realized reading output is reduced to approximately two thirds of its baseline value.

The constants 0.65 and 0.35 are heuristic rather than empirically fitted. They implement a bounded mechanical penalty: poor legibility can reduce realized reading output by at most 35%, while preserving a minimum residual reading efficiency of 65%. Future empirical work could replace this heuristic bound with reading-speed decrements estimated under controlled glare, contrast, and illuminance conditions.

## 4 Simulation

Active inference distinguishes between the **generative process**—the true dynamics of the person's cognitive state—and the **generative model**—the agent's internal probabilistic representation. In our model, at every step *t*, these two levels unfold in sequence, as the structure of Algorithm 1 shows.

Steps 3–4 and step 10 operate on different objects: the agent updates the probability distribution *Q*(*s*_*t*_); the environment updates the single sampled state $s_{t+1}^{*}$. Three consequences follow.

The agent can be wrong about its own state. When both *ϕ*_text_ and *ϕ*_eye_ are low (S3/center, *ϕ*_text_ = 0.047, and *ϕ*_eye_ = 0.002), both observation streams are nearly uninformative. *Q*(*s*_*t*_) remains close to the prediction ${\hat{\text{s}}}_{t}$ regardless of what $s_{t}^{*}$ actually is. The two-modality design does not eliminate this blind spot—it relocates it. At S3/center, glare is severe enough to compromise *both* signals simultaneously.

Wpm is determined by the true state, not the belief. $\text{wpm}\left(t\right)=r\left(s_{t}^{*}\right)\cdot \rho \left(u_{t}\right)\cdot \ell_{\text{factor}}$ depends on $s_{t}^{*}$, not on *Q*(*s*_*t*_). The legibility factor ℓ_factor_ is fixed by the scenario and position, not by the agent's inference.

The agent does not optimize wpm. It minimizes EFE. High wpm is a consequence of being focused, choosing to continue, and being in good light—not an objective the agent pursues.

This cycle repeats for *T* = 15 steps, spanning a 5-h morning session (09:00–14:00). Three factors are crossed in a 3 × 3 × 3 factorial design: lighting scenario (S1, S2, and S3), chronotype (lark, intermediate, and owl), and spatial position in the room (near_source, center, far_corner). The simulation is replicated *N*_MC_ = 20 times per condition (Number of Monte Carlo simulations).

As mentioned, the simulation uses three lighting scenarios, denoted S1, S2, and S3. These scenarios are not intended to represent three isolated lighting variables, but three integrated environmental regimes combining illuminance level, correlated color temperature, and spectral context. They provide the environmental inputs from which the model derives local illuminance, scenario-induced arousal, and action-context priors.

S1 corresponds to a warm-dim condition. It is characterized by low and relatively uniform illuminance, warm spectral character, and low scenario-induced arousal. In the model, this condition is associated with under-arousal, *a*_*base*_ ≈ 0.32, and therefore increases drift from focus toward distraction. S2 corresponds to a moderate neutral condition. It represents the reference lighting environment: illuminance is near the legibility optimum, the spectral context is neutral, and scenario-induced arousal is close to the model's neutral point, *a*_*base*_ ≈ 0.78. S3 corresponds to an intense bright-cool condition. It combines high illuminance near the lighting sources with a cool or blue-enriched spectral character and high scenario-induced arousal. In the model, this condition is associated with over-arousal, *a*_*base*_ ≈ 1.00, and therefore increases drift from focus toward fatigue.

The full source code used to implement the model and reproduce the simulations, analyses, and figures reported is available in the accompanying GitHub repository: https://github.com/DesignAInf/LIGHTING—ACTIVE—INFERENCE—PROJECT.

## 5 Results

The three-channel hypothesis is the theoretical claim of this paper; the six predictions P1–P6 are its operational, testable consequences and are, in that sense, the primary hypotheses the simulations evaluate. Each prediction isolates one structural commitment of the architecture: P1 tests the independence of the epistemic channel from chronotype; P2 tests the affective sign-reversal between chronotypes; P3 and P4 test the interaction of the epistemic and affective channels across space; P5 tests the chronotype-by-position interaction through the preference structure; and P6 tests the structural invariance implied by setting the intermediate tolerance factor to zero. These six predictions are the hypotheses under test, and they fall into three tiers by evidential status: structural invariants that verify the implementation (P1, P2, and P6), the critical test of the hypothesis (P3, P4), and one exploratory prediction (P5).

### 5.1 Falsifiable predictions

Six predictions are derived from the structure of the model. Each identifies the component being tested, the expected direction, and the result that would falsify it. Predictions P1 and P2 test the channels in isolation. P3 and P4 test their interaction through the spatial structure. P5 and P6 test predictions that require the full session to emerge.

#### 5.1.1 P1—Epistemic invariance across chronotypes

This prediction says that, if desk position and lighting scenario are held fixed, chronotype alone should not change how informative the two observation channels are. In the model, the quality of self-monitoring depends on local illuminance, not on whether the reader is a morning type, an intermediate type, or an evening type. So, at the same desk under the same lighting, all three chronotypes should have essentially the same level of uncertainty about their own state. If one chronotype systematically had clearer or noisier self-monitoring than the others, the model would no longer preserve the intended separation between the epistemic channel and the affective channel.

**A**_perf_(ϕ_text_) and **A**_eye_(ϕ_eye_) depend only on the illuminance at the agent's position, not on chronotype. Ambiguity is therefore chronotype-blind at any fixed position and scenario.

**Prediction**. The maximum ambiguity gap across chronotypes must remain below 0.10 nat at any fixed position and scenario.

A systematic ambiguity difference between chronotypes would indicate contamination of the epistemic channel by the affective channel.

#### 5.1.2 P2—Risk sign-reversal between lark and owl

This prediction says that the same lighting condition should not feel equally costly to all chronotypes. In bright-cool light (S3), morning types are pushed away from their preferred level of activation and should therefore evaluate poor performance more negatively, which raises their risk. Evening types should show the opposite pattern, because that same condition moves them closer to their preferred level of activation and makes low performance less aversive. In warm-dim light (S1), the direction reverses. So the model predicts a true sign-reversal: the condition that is riskier for the morning type should be less risky for the evening type, and vice versa.

κ_lark_ = −3.0 and κ_owl_ = +3.5 have opposite signs. Under S3 (*m* > 0, over-arousal), the lark's *C*_low_ decreases (higher risk) while the owl's increases (lower risk). The directions reverse under S1 (*m* < 0).

**Prediction**. ${\bar{R}}_{\text{lark}}\left(\text{S3}\right)>{\bar{R}}_{\text{lark}}\left(\text{S1}\right)$ and ${\bar{R}}_{\text{owl}}\left(\text{S1}\right)>{\bar{R}}_{\text{owl}}\left(\text{S3}\right)$.

Absence of the sign-reversal would indicate that the tolerance mechanism in **C** is not producing genuine affective asymmetry.

#### 5.1.3 P3—Spatial ordering under S2 follows the legibility gradient

This prediction concerns the moderate scenario (S2). In that condition, the model sets the arousal-related drift terms to zero, so desk position does not change the underlying state dynamics. What still varies across desks is the quality of the visual information available to the agent. For that reason, session performance should follow a simple spatial ordering: the center desk should do best, the near-source desk should come next, and the far-corner desk should do worst. Under S2, then, better legibility should translate directly into better self-monitoring and better reading output.

Under S2, Yerkes–Dodson drift terms are both zero (δ_under_ = δ_over_ = 0), so state dynamics in **B** are identical across positions. The only source of spatial variation is the epistemic channel.

**Prediction**. Under S2: ${\bar{\text{wpm}}}_{\text{center}}>{\bar{\text{wpm}}}_{\text{near\_source}}>{\bar{\text{wpm}}}_{\text{far\_corner}}$ for all three chronotypes.

Any reversal of the ordering, in particular if near_source outperforms center.

#### 5.1.4 P4—Spatial reversal

Under intense cool light (S3), the same spatial ordering found in S2 should flip. The far-corner desk should outperform the center desk, even though it is less intensely lit. The reason is that, at the far corner, the agent can still monitor its condition reasonably well and can rest strategically when deterioration becomes clear, while paying almost no direct glare cost on the reading itself. At the center desk, by contrast, glare suppresses both observation channels, so the agent has poor access to its own deteriorating state and keeps working with little awareness of the problem. At the same time, each working step is mechanically penalized by glare. The prediction is therefore not just that the desks differ, but that the full ranking reverses: under S3, far-corner should outperform near-source, and near-source should outperform center.

Under S2, the Yerkes–Dodson drift terms are both zero (δ_under_ = δ_over_ = 0), so state dynamics in **B** are identical across positions and the epistemic channel alone drives spatial variation. The result is a monotonic ordering that follows the legibility gradient (P3). Under S3, two mechanisms operate jointly and in the same direction at the better-lit positions:

1. *Epistemic mechanism*. At far_corner (ϕ_text_ = 0.941, *ϕ*_eye_ = 0.400), both observation channels are informative. The agent accurately perceives its predominantly deteriorated state—a direct consequence of the over-arousal drift in **B** (δ_over_ > 0 at *a* ≈ 1.00). Accurate self-monitoring increases risk and drives more frequent pausing. When not pausing, the mechanical legibility factor is ℓ_factor_ ≈ 1.0.
2. *Mechanical mechanism*. At center (ϕ_text_ = 0.047, *ϕ*_eye_ = 0.002), both observation channels are suppressed. The agent is blind to its own deterioration, pauses less, and keeps working—but the severe glare imposes a direct speed penalty of ℓ_factor_ = 0.666, reducing all output by one third regardless of cognitive state or action chosen.

At far_corner/S3, strategic rest combines with a negligible mechanical penalty. At center/S3, blind persistence combines with a severe mechanical penalty. The net result is a complete inversion of the spatial ordering relative to S2.

**Prediction**. Under S3, the spatial ordering of session wpm must be the reverse of the S2 ordering:


$$\begin{array}{l} {\bar{\text{wpm}}}_{\text{far\_corner}}>{\bar{\text{wpm}}}_{\text{near\_source}}>{\bar{\text{wpm}}}_{\text{center}}. \end{array}$$

This must hold for all three chronotypes. The EFE decomposition must additionally confirm that far_corner/S3 has *lower* total ambiguity and *higher* risk than center/S3—establishing that the better output at far_corner occurs despite, not because of, the agent's more demanding decision-making.

If the spatial ordering under S3 replicates the ordering under S2 (center best, far_corner worst), the interaction between the epistemic and affective channels—mediated by the mechanical legibility factor— is not producing the predicted inversion. This would suggest that the two channels operate independently at the level of behavioral output, which would require revising the architecture.

This prediction is not derivable from a single-channel model. A model in which lighting affects only arousal predicts position independence, since arousal is determined by the scenario, not by desk location. A model in which lighting affects only legibility predicts the same ordering under both S2 and S3—better legibility always produces better performance. Only the multi-channel architecture, in which the epistemic channel determines how accurately the agent perceives a state that the affective channel has made predominantly bad, predicts the inversion. P3 and P4 are therefore the joint critical experiment for the three-channel hypothesis: the same model that predicts a monotonic gradient under S2 must predict its reversal under S3.

#### 5.1.5 P5—Position-by-chronotype asymmetry under S3

This prediction adds a chronotype-by-position interaction. Under S3, the far-corner desk gives the agent good enough information to detect deterioration. That should help evening types more than morning types. The reason is not that evening types see better, but that they tolerate the over-aroused condition better once they detect it. As a result, they can use the good epistemic conditions of the far-corner desk without responding too defensively. Morning types, in contrast, should react more strongly to the same evidence of deterioration and pause more aggressively. So the gain from moving from center to far-corner should be much larger for evening types than for morning types.

Policy precision γ(*t, c*) is position-independent—it depends on *a*_base_ and a^*^(*t, c*), neither of which varies with desk location. However, the tolerance factor κ_*c*_ in **C**(*t, c*) modulates how the agent evaluates its own deteriorating state when that state becomes visible through the observation channels.

Under S3 (*m* > 0, over-arousal), the owl's κ_owl_ = +3.5 increases *C*_low_, reducing its aversion to low_perf observations. At far_corner/S3, where both channels are informative and accurately reveal deterioration, the owl's reduced aversion translates into lower risk and less aggressive pausing: it exploits far_corner's epistemic advantage efficiently. The lark's κ_lark_ = −3.0 does the opposite: it decreases *C*_low_, sharpening aversion to low performance. At far_corner/S3, the same accurate self-monitoring that benefits the owl triggers more aggressive pausing for the lark, limiting the productive use of the position's good legibility. The owl additionally carries a higher policy precision (γ_owl_ = 4.85 vs γ_lark_ = 3.88 under S3), making it more efficient at exploiting the EFE signal wherever it exists.

**Prediction**. Under S3, the wpm advantage of far_corner over center must be substantially larger for the owl than for the lark:


$$\begin{array}{l} {\left({\bar{\text{wpm}}}_{\text{far\_corner}}-{\bar{\text{wpm}}}_{\text{center}}\right)}_{\text{owl}}\\ \gg {\left({\bar{\text{wpm}}}_{\text{far\_corner}}-{\bar{\text{wpm}}}_{\text{center}}\right)}_{\text{lark}}. \end{array}$$

Equal far_corner advantages for lark and owl would indicate that κ_*c*_ is not modulating the response to accurate self-monitoring as predicted—that tolerance and precision interact independently of spatial structure.

#### 5.1.6 P6—Intermediate chronotype has lowest risk variance

This prediction says that the intermediate chronotype should be the most stable across S1, S2, and S3. In the model, this is a structural consequence of setting the intermediate type's affective tolerance factor to zero. Because of that, changes in the lighting scenario do not shift its evaluation of low performance in the same way they do for the morning and evening types. The result should be a much smaller change in risk across scenarios. So the prediction is not simply that the intermediate type performs “in the middle,” but that it is the least scenario-sensitive in terms of risk.

With κ_int._ = 0, the intermediate's $C_{\text{low}}\left(t,c\right)=C_{\text{low}}^{\text{base}}$ is not modulated by the arousal mismatch. Its risk is therefore structurally invariant across scenarios by construction. Lark and owl, with non-zero κ, show large risk swings between S1, S2, and S3.

**Prediction**. ${\text{Var}}_{S\in \left\{S1,S2,S3\right\}}\left[{\bar{R}}_{\text{int.}}\left(S\right)\right]<\text{Var}\left[{\bar{R}}_{\text{lark}}\left(S\right)\right]$ and $<\text{Var}\left[{\bar{R}}_{\text{owl}}\left(S\right)\right]$.

Intermediate risk variance comparable to the extreme chronotypes, implying that κ = 0 is not sufficient to insulate the agent from scenario-induced preference shifts.

### 5.2 Analysis results

All 27 conditions were simulated with *N*_MC_ = 20 replicates each. All six predictions are confirmed. Table 4 provides a compact summary; the subsections below discuss each in detail.

**Table 4:** Prediction outcomes (*N*_MC_ = 20, center position unless noted).

ID	Prediction	Key result at *N*_MC_ = 20
P1	Epistemic invariance	Chronotype ambiguity gap 0.0014 nat at S2 (threshold 0.10); below 0.10 nat at every position and scenario.
P2	Risk sign-reversal	Lark risk 0.89 → 1.52 (S1 → S3) while owl 1.35 → 0.62; the two move in opposite directions.
P3	Spatial ordering, S2	center > near_source > far_corner for all chronotypes (intermediate 79.2 > 73.0 > 61.0 wpm).
P4	Ordering inverts, S3	far_corner > near_source > center for all chronotypes (MC mean 71.3 > 63.7 > 59.9 wpm).
P5	Position × chronotype, S3	far_corner−center gap: owl +16.2 vs. lark +8.1 wpm (about twice).
P6	Intermediate robustness	Risk variance 0.011 (intermediate) vs. 0.075 (lark) and 0.110 (owl); 6.8–9.9 × lower.

All six predictions are confirmed; the key quantitative result is reported for each.

#### 5.2.1 P1—Epistemic invariance across chronotypes

At S2/center (ϕ_text_ = 1.00, *ϕ*_eye_ = 0.40), the performance ambiguity gap across chronotypes is 0.0014 nat, more than two orders of magnitude below the 0.10 nat threshold, and it stays below 0.10 nat at every position and scenario tested, confirming P1. The near-zero gap confirms that the epistemic channel is operationally chronotype-blind.

#### 5.2.2 P2—Risk sign-reversal between lark and owl

Table 5 reports the mean session risk at the center position for each chronotype and scenario. The lark's risk nearly doubles from S1 to S3, while the owl's approximately halves (1.352 to 0.623)—the sign-reversal that P2 predicts.

**Table 5:** Mean session risk (nat) at center (*N*_MC_ = 20).

Chronotype	S1	S2	S3
Lark	0.885	1.023	**1.523**
Owl	**1.352**	0.679	0.623

Bold, highest-risk scenario per chronotype.

The lark's risk nearly doubles from S1 to S3; the owl's approximately halves (1.352 to 0.623), the sign-reversal that P2 predicts. The mechanism is the tolerance factor κ_*c*_: at S3 (*m* > 0, over-arousal), the lark's *C*_low_ decreases (heightened aversion) while the owl's increases (reduced aversion). The directions reverse at S1 (*m* < 0).

#### 5.2.3 P3—Spatial ordering under S2

**Figure 3:**
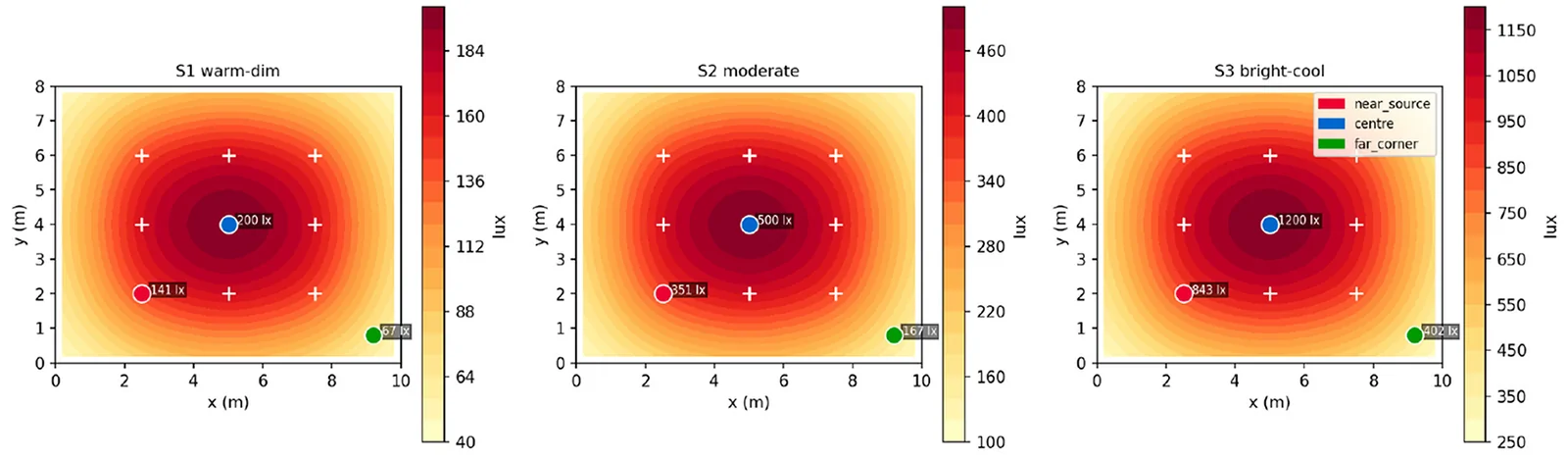
Photometric floor maps for the three lighting scenarios. Color encodes illuminance in lux; white crosses mark ceiling fixture positions; colored dots mark the three agent desk positions with their effective illuminance values. The spatial structure determines both precision functions:at S3, the far-corner position receives 402 lux (ϕ_text_ = 0.941, *ϕ*_eye_ = 0.400), while the center position receives 1,200 lux (ϕ_text_ = 0.047, *ϕ*_eye_ = 0.002). This 20-fold difference in legibility precision, combined with the affective channel's over-arousal drift at S3, drives the complete inversion of the spatial performance ordering examined in P4: the far corner—furthest from the fixtures—outperforms the center under intense cool light, despite being the worst-performing position under moderate light (see P3 and Table 6).

**Figure 4:**
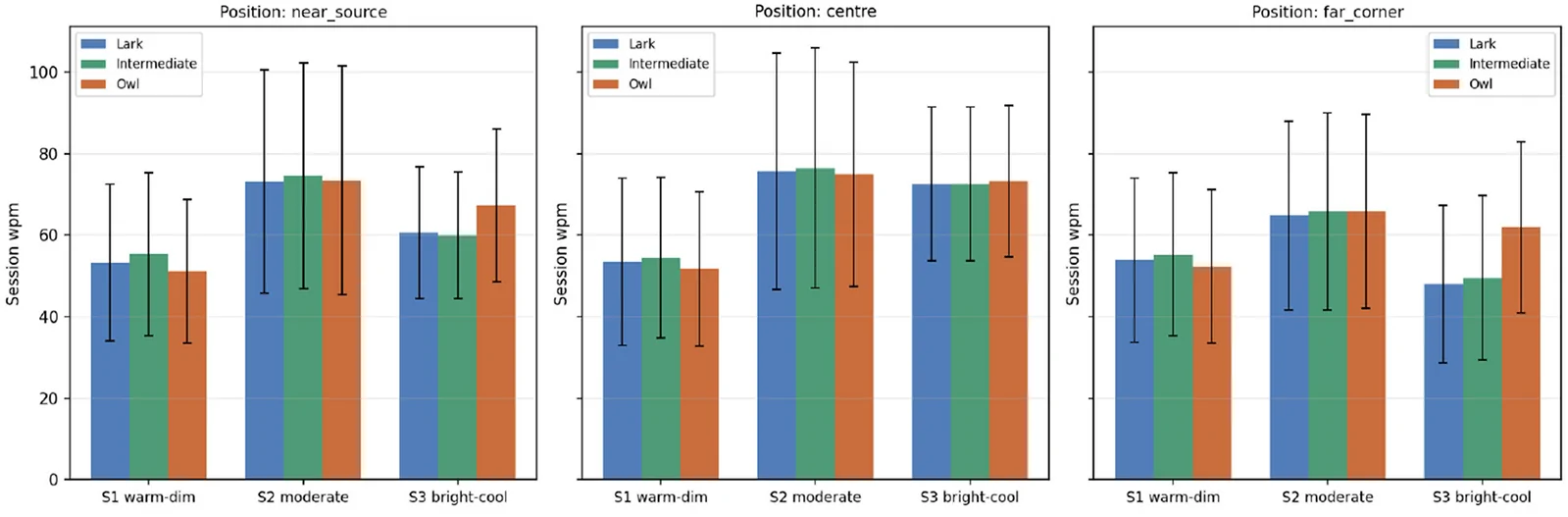
Session wpm by lighting scenario, chronotype, and spatial position (*N*_MC_ = 20; error bars are ±1 SD across replicates). Under S2 **(center)**, the spatial ordering center > near_source > far_corner holds consistently for all three chronotypes (P3): the legibility gradient maps directly onto performance. Under S3 **(right)**, this ordering is completely reversed: far_corner > near_source > center (P4). The position that performed worst under moderate lighting performs best under intense cool lighting. Under S1 **(left)**, scenario-level differences are modest and spatial variation is small, reflecting the low and nearly uniform illuminance across all positions. P3 and P4 together constitute the critical experiment for the three-channel hypothesis (see Section 5.1).

The photometric floor maps in Figure 3 give the effective illuminance at each of the three desk positions under the three scenarios. The center panel of Figure 4 shows the resulting session wpm under S2, which follows the legibility gradient for all three chronotypes. Table 6 reports session wpm under S2 by chronotype and position.

**Table 6:** Session wpm under S2 by chronotype and position (*N*_MC_ = 20).

Chronotype	center	near_source	far_corner
Lark	**77.3**	71.4	59.9
Intermediate	**79.2**	73.0	61.0
Owl	**82.8**	75.1	64.8

Bold type marks the value of principal interest for the comparison shown: the highest session reading speed per chronotype under S2.

**Figure 5:**
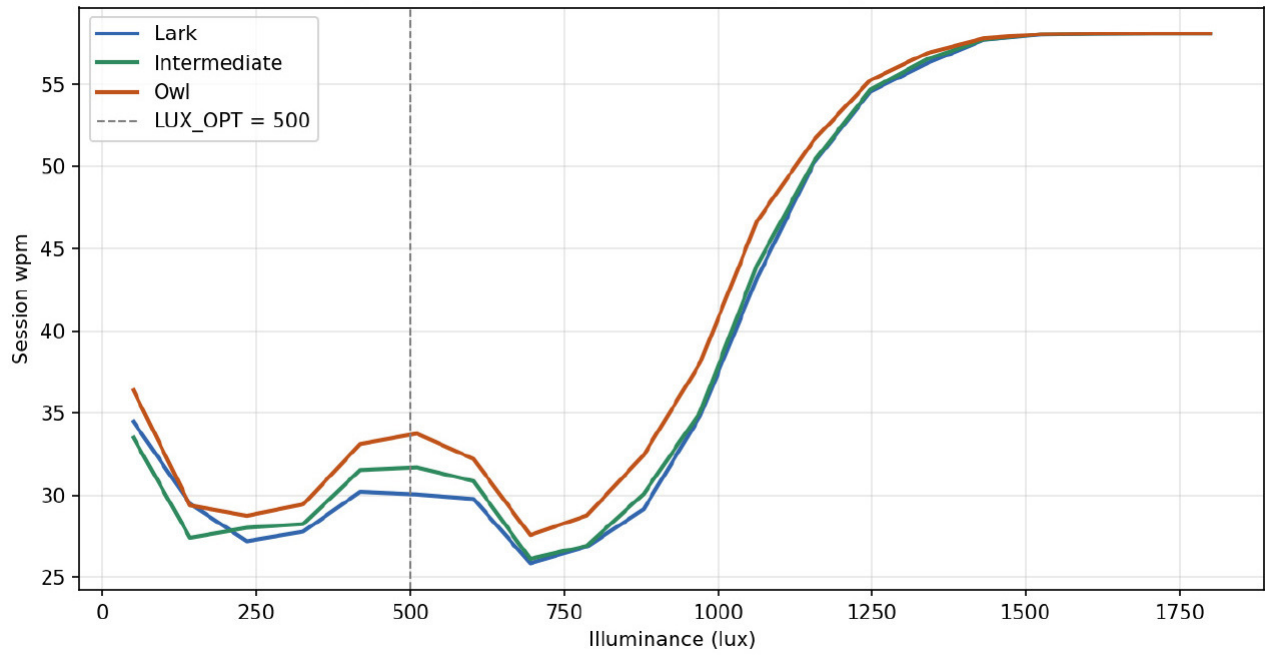
Continuous lux sweep: session wpm as a function of illuminance S2 scenario, center position, *n* = 5 seeds per lux level. The inverted-U shape reflects the text legibility precision function *ϕ*_text_(ℓ): wpm rises steeply from 50 lux to the optimum at ℓ^*^ = 500 lux (dashed line), then declines more gradually as glare accumulates. The asymmetric slope—steeper on the under-illumination side than on the glare side—reflects the mechanical legibility factor (ℓ_factor_ = 0.65+0.35ϕ_text_), which sets a floor on reading speed even at extreme illuminance. Chronotype differences are visible primarily around the optimum, where precision differences in γ(*t, c*) amplify the EFE-minimizing advantage of a well-matched arousal state.

The spatial ordering center > near_source > far_corner holds for all three chronotypes (intermediate: 79.2>73.0>61.0 wpm), confirming P3. Under S2, with zero Yerkes–Dodson drift, the epistemic channel alone determines spatial variation: better legibility produces better self-monitoring and more efficient action selection. Figure 5 reports a continuous illuminance sweep at the center position under S2, in which session wpm traces the inverted-U induced by the text-legibility precision function.

#### 5.2.4 P4—Spatial ordering inverts under S3

As shown in the right panel of Figure 4, the S2 ordering is reversed under S3; Table 7 reports the underlying wpm values and EFE decomposition.

**Table 7:** Session wpm and EFE decomposition under S3 (MC mean across chronotypes, *N*_MC_ = 20).

Position	ϕ_text_	ϕ_eye_	ℓ_factor_	wpm	Pause%	Amb/Risk
far_corner	0.941	0.400	0.979	**71.3**	32%	1.805/1.263
near_source	0.479	0.108	0.818	63.7	29%	2.106/1.154
center	0.047	0.002	0.666	59.9	18%	2.197/1.063

The spatial ordering is completely inverted relative to S2 (Table 6). ℓ_factor_ = 0.65+0.35ϕ_text_. Bold type marks the value of principal interest for the comparison shown: the highest session reading speed across positions under S3.

P4 holds for all three chronotypes: the spatial ordering under S3 is the exact reverse of the S2 ordering, with far_corner best and center worst (lark 67.6/60.0/59.5; intermediate 69.8/62.6/59.9; owl 76.4/68.5/60.3 wpm).

The EFE decomposition reveals the two-part mechanism. At far_corner/S3: both precision values are substantial (ϕ_text_ = 0.941, *ϕ*_eye_ = 0.400); total ambiguity is low (1.805 nat); risk is high (1.263 nat), because accurate self-monitoring reveals a predominantly deteriorated cognitive state; the agent pauses 32% of steps. But when it does work, the mechanical legibility factor is ℓ_factor_ = 0.979—virtually no glare tax. At center/S3: both precision values are near zero (ϕ_text_ = 0.047, *ϕ*_eye_ = 0.002); total ambiguity is high (2.197 nat); risk is moderate (1.063 nat), because the near-flat **A** matrices make all predicted observations nearly uniform, insensitive to the true (bad) state; the agent pauses only 18% of steps. But every working step is penalized: ℓ_factor_ = 0.666, reducing all output by one third.

The inversion arises from two forces operating in the same direction at far_corner: strategic rest (avoiding wasted effort in a bad state) combined with mechanical efficiency (each working step is nearly penalty-free). At center, the opposite forces combine: blind persistence (ignoring the bad state) combined with mechanical penalty (taxing every working step). The position that supports strategic rest without imposing a severe mechanical glare penalty produces more output.

This result requires the multi-channel architecture for two distinct reasons. First, the ambiguity-risk dissociation across positions—low ambiguity and high risk at far_corner, high ambiguity and moderate risk at center—requires the epistemic channel to modulate self-monitoring quality by position. Second, the mechanical legibility penalty requires the epistemic channel to act directly on output speed per working step. Neither effect is present in an arousal-only model, where desk position is irrelevant. Together, P3 and P4 constitute the critical experiment: the same epistemic channel that produces a monotonic gradient under S2 (affective channel neutral) produces a complete reversal under S3 (affective channel active).

#### 5.2.5 P5—Position-by-chronotype asymmetry under S3

**Figure 6:**
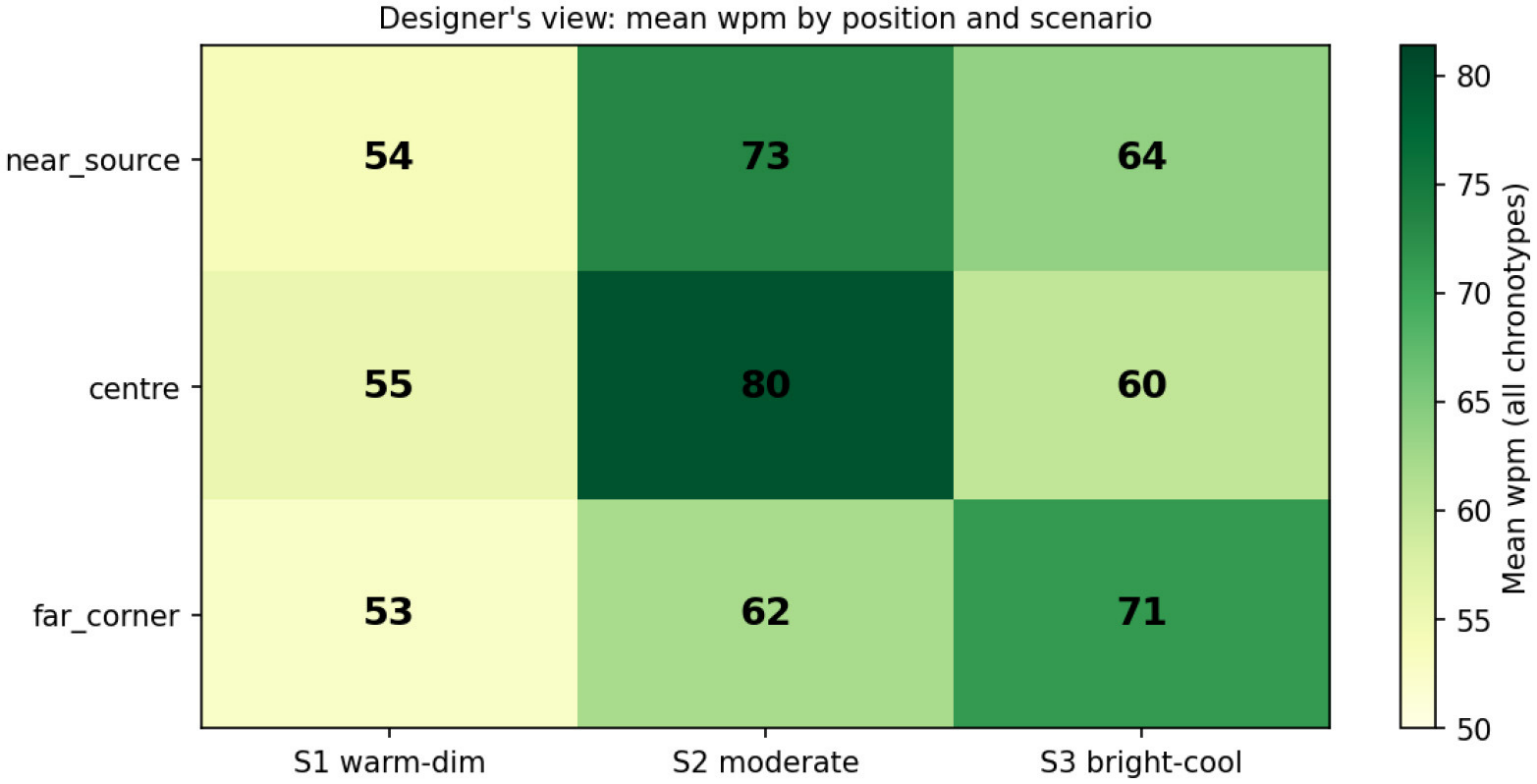
Designer's view: mean session wpm averaged across all three chronotypes, by spatial position **(rows)** and lighting scenario **(columns)**. Numbers in each cell are wpm. The position-by-scenario interaction is clearly visible: under S2, the center position dominates; under S3, far-corner and near-source positions recover relative to center, with far-corner producing the highest population-level mean (≈71 wpm). The chronotype-specific asymmetry of this effect—the subject of P5—is not visible in this population-average view but is reported in Table 8.

Figure 6 gives the population-level view–mean session wpm by position and scenario, averaged across chronotypes. Table 8 makes explicit the chronotype asymmetry this average conceals, reporting the far_corner-over-center advantage under S3 for each type.

**Table 8:** Wpm advantage of far_corner over center under S3, by chronotype (*N*_MC_ = 20).

Chronotype	far_corner	center	Gap	*gamma*	Pause% at far
Owl	76.4	60.3	**+16.2**	4.85	25%
Intermediate	69.8	59.9	+9.9	4.93	33%
Lark	67.6	59.5	+8.1	3.88	36%

Positive gap means far_corner outperforms center. Bold type marks the value of principal interest for the comparison shown: the largest advantage of the far corner over the centre under S3.

P5 holds: the owl's far_corner advantage of +16.2 wpm is roughly twice the lark's +8.1 wpm. The mechanism operates through two concurrent effects of the tolerance factor κ_*c*_ under over-arousal (*m*>0 at S3).

At far_corner/S3, both observation channels are informative (ϕ_text_ = 0.941, *ϕ*_eye_ = 0.400) and the agent accurately perceives its deteriorating state. For the lark (κ = −3.0), this accurate perception is costly: sharper aversion to low_perf inflates risk to 1.771 nat and triggers aggressive pausing (36% of steps). For the owl (κ = +3.5), the same accurate perception is tolerated: reduced aversion keeps risk at 0.772 nat and pause rate at only 25%. The owl's higher policy precision (γ = 4.85 vs 3.88) amplifies this advantage by making it more efficient at exploiting the position's good epistemic conditions.

At center/S3, both channels are suppressed and the two chronotypes produce similar wpm (59.5 and 60.3, respectively): when neither agent can see its own state, the tolerance asymmetry has little consequence.

The gap therefore arises entirely from what happens at far_corner: the owl's arousal tolerance makes it better able to use accurate self-knowledge productively, while the lark's intolerance of over-arousal turns the same accurate information into a trigger for excessive rest.

#### 5.2.6 P6—Intermediate chronotype has lowest risk variance

**Figure 7:**
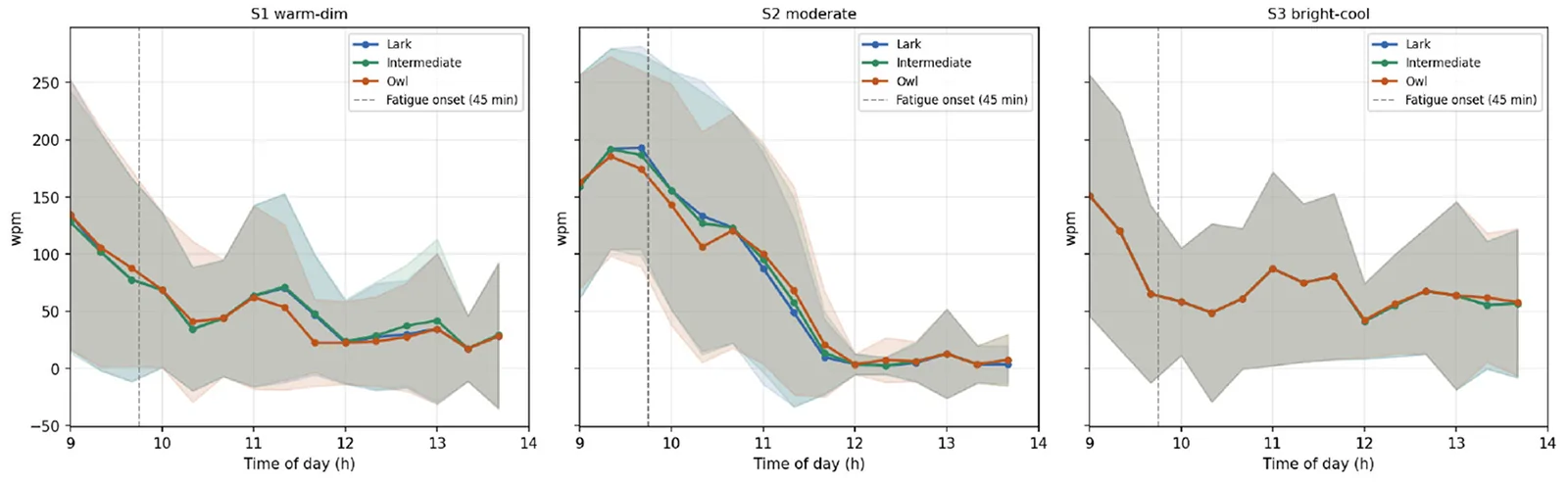
Running wpm over the *T* = 15 decision steps (MC mean ±1 SD, center position, *N*_MC_ = 20). Each panel shows one lighting scenario; colored lines correspond to chronotypes. The dashed vertical line marks the fatigue onset at 45 mins (step 3). In both S1 **(left)** and S3 **(right)**, the lark–owl ordering is absent or reversed in the first half of the session and settles into its final direction only in the second half, consistent with P6. The large step-to-step variance reflects the stochastic nature of the true-state transitions and the coarse 20-min temporal resolution.

**Figure 8:**
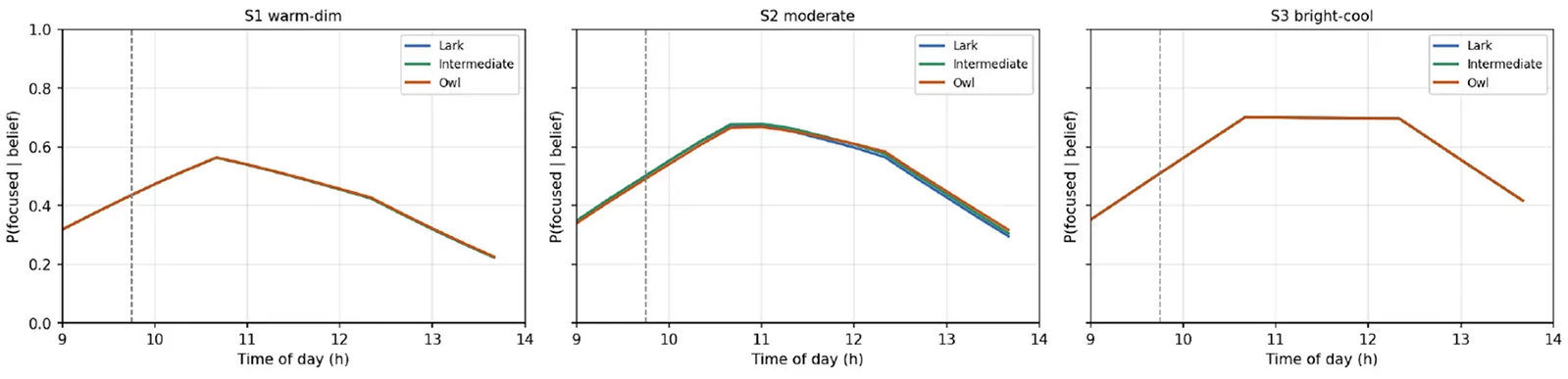
Posterior belief *Q*(*s* = focused) over the session (MC mean, center position, *N*_MC_ = 20). The dashed vertical line marks the fatigue onset at 45 mins (step 3). Three phases are distinguishable across all conditions: a stable high-belief phase [steps 0–2, *f*(*t*) = 1.00]; a progressive decline phase [steps 3–8, *f*(*t*) falling from 0.93 to 0.46]; and a near-collapsed phase [steps 9–14, *f*(*t*) ≤ 0.34]. Under S3/center, where both observation channels are suppressed (ϕ_text_ = 0.047, *ϕ*_eye_ = 0.002), the posterior remains close to the prior **D** throughout, compressing confidence bands and masking the genuine deterioration of the true state.

Figure 7 shows the running wpm across the fifteen decision steps for each scenario and chronotype, with the fatigue onset at 45 mins marked by the dashed line: the lark-owl ordering emerges only in the second half of the session. What remains stable across scenarios, rather than over time, is reported in Table 9, which gives the variance of mean session risk across S1-S3 for each chronotype. The mechanism behind both patterns is visible in Figure 8, which traces the posterior belief Q(s = focused) over the session and shows how glare at S3/center keeps the posterior close to the prior D, masking the genuine deterioration of the true state.

**Table 9:** Variance of mean session risk across scenarios S1–S3 (center position, *N*_MC_ = 20).

Chronotype	Risk variance
Lark	0.0750
Intermediate	**0.0111**
Owl	0.1098

Bold type marks the value of principal interest for the comparison shown: the lowest variance in session risk across scenarios.

P6 holds: the intermediate's risk variance of 0.011 is 6.8 × lower than the lark's and 9.9 × lower than the owl's. This is a structural invariant of the model: with κ_int._ = 0, the mismatch *m*(*t, c*) does not shift *C*_low_, making risk independent of scenario by construction. This prediction would survive any reparametrization of the model as long as κ_int._ = 0 is maintained.

#### 5.2.7 Fatigue as a session-level moderator

An unexpected finding concerns the relative wpm decline from the first to the second half of the session:


$$\begin{array}{l} \Delta {\bar{\text{wpm}}}_{\text{S1}}\approx 34,\;\Delta {\bar{\text{wpm}}}_{\text{S2}}\approx 100,\;\Delta {\bar{\text{wpm}}}_{\text{S3}}\approx 15. \end{array}$$

The decline is largest under S2 and smallest under S3. The mechanism is the same as P4: under S2 (ϕ_text_ = 1.00, *ϕ*_eye_ = 0.40), the agent accurately perceives its progressive fatigue and responds with appropriate but output-reducing rest. Under S3/center (ϕ_text_ = 0.047, *ϕ*_eye_ = 0.002), both signals are blind to fatigue; the agent keeps working but at reduced speed due to ℓ_factor_. The two-modality design makes the fatigue buffering effect of glare visible at a finer level: not only does the agent fail to perceive fatigue, it fails across *both* observation channels simultaneously, leaving it with no route to accurate self-assessment.

## 6 Summary of results

**Figure 9:**
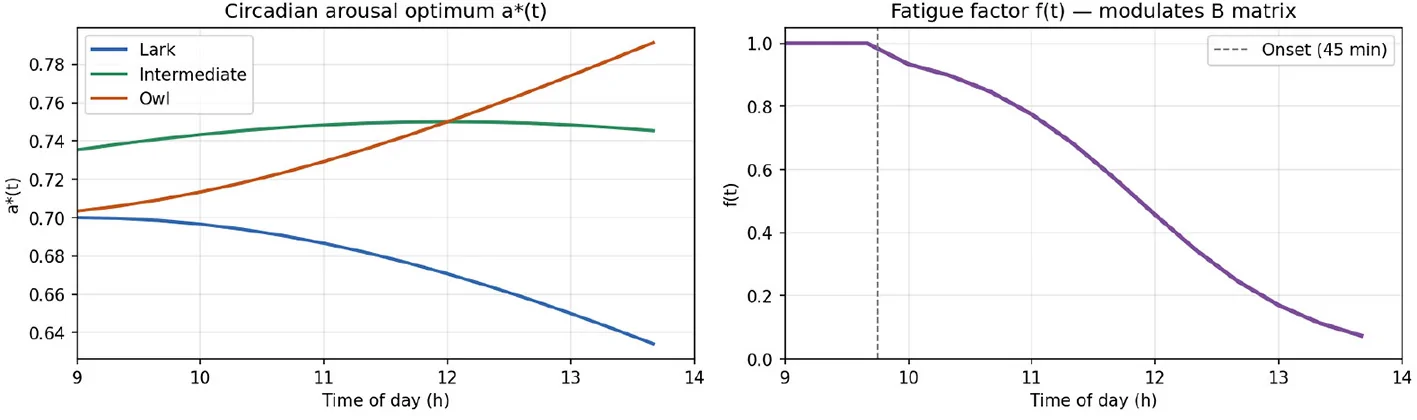
**(Left)** Circadian arousal optima a^*^(*t, c*) for the three chronotypes over the 5-h session (09:00–14:00). The lark (blue) declines from 0.70 at session onset to 0.63 by 14:00; the owl (orange) rises from 0.70 to 0.80; the intermediate (green) follows a shallow arc peaking near noon. The 0.17-unit divergence between lark and owl by session end drives the half-session crossover. **(Right)** Fatigue factor *f*(*t*). Full attentional capacity (*f* = 1.00) is maintained for the first 45 mins; thereafter *f* follows a sigmoid decay, reaching *f* = 0.46 at 160 mins and *f* = 0.07 at the end of the session. The fatigue factor modulates **B**(*t, a*) via linear interpolation between the fresh and tired transition matrices.

The simulation supports the three-channel hypothesis and yields a coherent set of results. Figure 9 shows the two time-varying components that drive these effects: the circadian optima a^*^(*t, c*) and the fatigue factor *f*(*t*).

The epistemic channel is invariant across chronotypes: at any fixed desk position and lighting scenario, morning, intermediate, and evening types receive the same quality of information about their own state. By contrast, the affective channel produces a sign-reversal between morning and evening types: bright-cool light increases risk for morning types and decreases it for evening types, while warm-dim light produces the opposite pattern. Under moderate light (S2), performance follows the expected legibility gradient, with center outperforming near-source and far-corner. Under intense cool light (S3), however, this ordering reverses: far-corner outperforms center because it preserves informative observation signals and imposes little glare penalty, whereas center suppresses both channels and mechanically slows reading. This advantage is larger for evening types than for morning types, while the intermediate chronotype remains the most stable across scenarios because its preference structure is less sensitive to arousal mismatch. An additional unexpected result concerns fatigue over time: the decline in reading speed is greatest under S2 and smallest under S3. The reason is that, under S2, the agent can accurately detect fatigue and responds by resting more, whereas under S3 glare suppresses both observation channels, so the agent continues working despite deteriorating performance. In this sense, glare acts as an involuntary fatigue buffer: not by improving functioning, but by reducing access to the evidence of decline.

## 7 Objections and replies

### 7.1 The predictions are *post-hoc* rationalizations of simulation outputs

An objector may argue that the six predictions were formulated only after the model had already been built and simulated, and therefore amount to redescriptions of the output rather than genuine predictions.

#### 7.1.1 Reply

This objection misstates the logic of the paper. The predictions are not summaries of whatever the simulation happened to produce. They follow from specific structural commitments made when the generative model was defined. The derivational order is: hypothesis, architecture, prediction, simulation. The role of the simulation is to verify that those architectural commitments generate the expected qualitative signatures.

This distinction matters because each prediction probes a different part of the model. P1 tests whether the epistemic channel remains independent of chronotype. P2 tests whether the affective channel generates the expected reversal between morning and evening types. P3 and P4 test whether epistemic and affective mechanisms interact in a way that produces both a monotonic spatial gradient under S2 and its reversal under S3. P5 tests whether chronotype and spatial position interact through the preference structure. P6 tests whether setting κ_int._ = 0 is sufficient to make the intermediate chronotype comparatively invariant across scenarios. In each case, a failure would implicate a specific structural commitment rather than a merely cosmetic choice of wording.

### 7.2 The parameters are calibrated to make the predictions come out right

A second objection is that several parameter values are not empirically estimated and may have been chosen so that the six predictions hold. On this view, the model shows only that one internally coherent parametrization exists.

#### 7.2.1 Reply

This objection is substantially correct, and the paper does not deny it. The present model is not offered as an empirically calibrated account of human reading performance. It is a proof of concept designed to show that the three-channel hypothesis can be implemented as a coherent generative architecture that yields distinct, falsifiable predictions. At this stage, the main question is not whether the chosen values are already correct in an empirical sense, but whether the architecture is tractable and whether its qualitative behavior is robust enough for later calibration to be meaningful.

For that reason, the strongest support at this stage comes from structural robustness rather than empirical fit. Appendix C shows that the model's main qualitative signatures remain interpretable across plausible variations of several key parameters. In addition, some modeling choices are not introduced as fitted values at all, but as theoretical commitments. The intermediate chronotype is assigned κ_int._ = 0 because the model treats it as having no systematic arousal-tolerance asymmetry. The text-legibility precision function is symmetric because there is no clear reason to assume that dim light and glare should affect text legibility asymmetrically in the same way as the oculomotor signal. By contrast, the eye-tracking precision function is asymmetric because glare has a specific physiological effect through pupil constriction that is not mirrored under low illuminance. These choices are therefore open to empirical challenge, but they are not merely tuned to rescue the model.

### 7.3 The three channels are not structurally independent

A third objection is that the channels are not genuinely distinct because they share upstream physical drivers. In particular, the spectral coefficient μ(σ) contributes both to the affective channel, through baseline arousal, and to the normative channel, through the policy prior.

#### 7.3.1 Reply

This is a real structural feature of the model, but it is not a defect. The channels are distinguished by the component they modify and by the computational role that component plays in inference and action selection, not by complete independence of physical input. A single environmental variable can affect what the agent can observe, what the agent prefers, and what the agent is disposed to do without collapsing those functions into a single mechanism.

For this reason, the relevant test of channel distinctness is not input purity but selective ablation. If one removes the spectral contribution to arousal while leaving the normative prior intact, the affective signature should weaken while the normative effect should remain. That is the relevant sense in which the channels are structurally distinct: they can be functionally dissociated even when they are not driven by wholly separate physical variables.

Under the metrology adopted in this paper, this point can be sharpened. Because the affective channel is indexed by melanopic EDI and the normative channel by chromaticity, the two are dissociable by metamerism: a multi-primary source can hold chromaticity fixed while varying melanopic EDI, and conversely. The channels are therefore separable not only in computational role but, under such a manipulation, in physical input as well; their covariation in ordinary luminaires is a contingent hardware fact rather than a structural identity.

### 7.4 A simpler, single-channel model would explain the same results

An objector may further claim that the three-channel architecture is unnecessary. A suitably chosen one-factor model, for example an arousal-only model, might reproduce the main scenario-by-chronotype pattern more parsimoniously.

#### 7.4.1 Reply

The strongest response is the joint evidence from P3 and P4. In S2, where the affective channel is neutral, performance follows legibility: center performs best, then near-source, then far-corner. In S3, however, this ordering reverses, with far-corner outperforming center. A purely arousal-based model cannot explain position effects within the same scenario, while a purely legibility-based model would predict the same spatial ordering across scenarios. Neither predicts a pattern that is monotonic in S2 but reversed in S3.

The EFE decomposition confirms this. In S3, far-corner has lower ambiguity but higher risk, while center has higher ambiguity but lower risk. Thus, the better-performing position is not simply the one with the easier decision problem. Only the three-channel architecture captures this combination of improved self-monitoring, increased aversiveness, and reduced glare cost. It is therefore doing explanatory work that single-channel alternatives cannot easily reproduce.

### 7.5 The agent is cognitively unrealistic

Another objection is that the agent is too idealized. It plans ahead, updates beliefs with exact Bayesian inference, has no episodic memory or external distraction, and experiences fatigue only through a predetermined function.

#### 7.5.1 Reply

This objection is valid only if the model is read as a descriptive simulation of an actual human reader. That is not the claim of the paper. The model is normative: it specifies how an agent with a particular generative model and preference structure would behave under changing lighting conditions. In active inference, this idealization is useful rather than embarrassing, because it allows empirical deviations to become informative about what the model leaves out.

The simplifications are also not arbitrary. The planning horizon is meant to capture the timescale of decisions about continuing, rereading, or pausing. Policy precision already introduces a graded form of bounded rationality, since low precision makes action selection less tightly coupled to expected free energy. The addition of the oculomotor channel also weakens an important idealization present in simpler models: it does not assume that the agent always has access to a reliable self-performance signal. Under glare, even this second source of information degrades, introducing bounded observability into the model itself.

### 7.6 The model adds nothing to the Yerkes–Dodson curve

A final objection is that the model simply redescribes a familiar inverted-U relation between arousal and performance in more formal language.

#### 7.6.1 Reply

The Yerkes–Dodson curve is a descriptive regularity, not a computational architecture. It states that performance often peaks at intermediate arousal, but it does not explain which part of the decision process is affected, how self-monitoring works, how illuminance and arousal interact spatially, or how these effects vary over time and across chronotypes.

The present model goes beyond restating the inverted-U relation. It embeds arousal within a structure that distinguishes perceptual precision, affective preference, and normative action bias. This allows it to generate predictions that a simple arousal-performance curve cannot express. P4 is the clearest case: under certain spatial and affective conditions, better self-monitoring produces more rest and higher total output, whereas severe glare produces more persistence but lower output. A descriptive inverted-U curve has no observer model and no mechanism for this result. If confirmed empirically, the Yerkes–Dodson curve becomes a limiting case of a richer computational account, not a sufficient explanation by itself.

## 8 Discussion

A natural question is why active inference, specifically, is the right vehicle for this account, rather than a simpler stimulus–response or arousal–performance model. The answer is that active inference is, to our knowledge, the only framework in which a single environmental variable can act simultaneously on perception, evaluation, and action within one objective function, rather than through separately engineered modules. This is precisely what the three-channel hypothesis requires: the same lighting condition must be able to change how clearly the agent sees its own state, how it values the outcomes it observes, and which actions it is disposed to take.

Three features of the framework carry the argument. First, precision is the formal substrate of attention, which gives illuminance a principled place to act: the epistemic channel is not an ad hoc noise term but the modulation of likelihood precision, exactly the quantity active inference already uses to model sensory gain (Feldman and Friston, 2010). Second, the decomposition of expected free energy into ambiguity and risk is what produces the non-trivial result of P4, in which better self-monitoring leads to strategic rest and higher total output while blind persistence under glare leads to lower output. A descriptive arousal–performance curve has no observer model and therefore no way to express this; an arousal-only account predicts no effect of desk position at fixed scenario, and a legibility-only account predicts the same spatial ordering in every scenario. Only a model that separates self-monitoring quality from outcome evaluation predicts an ordering that is monotonic under moderate light and reversed under intense light.

Third, because behavior emerges from minimizing expected free energy rather than from maximizing performance, reading speed is an emergent consequence of inference under lighting rather than a fitted response. This is what allows the model to generate falsifiable, cross-measure dissociations—between performance and self-monitoring, between chronotypes, and across desk positions—rather than a single tunable curve. In this sense the familiar Yerkes–Dodson relation appears as a limiting case of a richer account: the model reproduces the inverted-U in aggregate while specifying which part of the decision process each lighting variable acts on, and how those effects unfold over time and space.

These strengths should be weighed against the framework's commitments. The model is normative rather than descriptive: it specifies how an agent with a given generative model and preferences would behave, so empirical deviations are informative rather than fatal, but the account is not a simulation of a particular reader. The parameters are illustrative and not empirically calibrated; their role, established here, is to show that the architecture is tractable and that its qualitative signatures are robust (Appendix C), which is the precondition for the empirical calibration set out in the Conclusions.

## 9 Conclusions

This paper develops a proof-of-concept active inference model of the cognitive and emotional effects of shared indoor lighting. Its main contribution is not to provide a calibrated account of human reading behavior, but to formalize the hypothesis that lighting shapes behavior through three computationally distinct but interacting channels: an epistemic channel affecting self-monitoring precision, an affective channel affecting the evaluation of performance under different arousal states, and a normative channel affecting action tendencies. In this sense, the value of the model lies less in any single numerical result than in showing that the three-channel hypothesis can be expressed as an explicit generative architecture with distinct, falsifiable behavioral signatures.

The most important next step is therefore empirical. The model specifies what should be measured—reading speed, oculomotor metrics, pause frequency, and subjective confidence—and under what conditions these measures become theoretically informative: prolonged reading sessions, multiple desk positions, different lighting scenarios, and mixed chronotype groups. On this view, the strongest test of the framework is not whether a single aggregate performance curve is replicated, but whether the predicted dissociations emerge across measures: for example, whether chronotypes differ in risk across scenarios, whether intermediate types are more stable than extreme chronotypes, and whether behavioral output diverges from self-monitoring under severe glare. If such signatures are confirmed, indoor lighting will need to be understood not only as a variable that changes performance, but also as a condition that shapes how agents perceive themselves, evaluate their own state, and regulate action over time.

These signatures connect directly to workplace-lighting practice. Standard provisions specify task-plane illuminance targets for office and reading work (e.g., EN 12464-1, see CEN, 2021), and office studies report that the benefits of higher illuminance are task- and time-of-day dependent rather than monotonic (Huiberts et al., 2015; Ru et al., 2021; Jung et al., 2024; Li and Yao, 2025). The present model offers a mechanism for these regularities and, more practically, reframes the design objective: because local illuminance intensity can mask cognitive decline—the fatigue-buffering effect of glare—a performance-based specification should preserve the informativeness of self-monitoring (adequate but non-glaring task light, and attention to the spatial distribution of luminance) rather than maximize illuminance at the task plane alone.

A natural and architecturally important extension is to endow the agent with spatial agency. This requires only augmenting the action set with a “relocate/reorient” action that changes the agent's position in the photometric field, and hence its local illuminance ℓ, making desk position a controllable state rather than a fixed condition. The agent would then trade the cost of moving against the expected epistemic and mechanical benefits of better light, turning the model from a passive-recipient account into an interactive, predictive design tool—for example, predicting when an occupant should move away from a glaring center position, exactly the P3/P4 inversion made actionable.

## Data Availability

The datasets presented in this study can be found in online repositories. The names of the repository/repositories and accession number(s) can be found below: https://github.com/DesignAInf/LIGHTING—ACTIVE—INFERENCE—PROJECT.

## Footnotes

1 The Yerkes-Dodson curve (Yerkes and Dodson, 1908) describes an inverted-U relationship between arousal and performance: too little arousal produces poor performance due to inattention, too much produces poor performance due to overload, and the optimum lies somewhere in between. The exact position of the peak depends on task difficulty—simpler tasks tolerate higher arousal, while complex tasks are impaired by it sooner.

2 Throughout the literature review we report findings in terms of CCT, because this is the descriptor the reviewed studies used. We note, however, that CCT is a lossy scalar summary of the spectral power distribution: two sources with the same CCT can differ in melanopic content and therefore in their non-image-forming, arousal-relevant effect. For this reason our own model (Section 3) indexes the affective channel not by CCT but by melanopic equivalent daylight illuminance (melanopic EDI), the metrologically appropriate descriptor of the melanopsin-mediated response (CIE S 026:2018; Chellappa et al., 2011 already observe that spectral composition captures variation that nominal CCT values only approximate).

3 The numerical values used in these precision functions should be understood as heuristic parameter choices. They are motivated by the literature reviewed above—especially the non-linear effects of illuminance on reading and the glare sensitivity of oculomotor signals—but they are not fitted estimates. Their role is to instantiate a plausible proof-of-concept architecture that can later be empirically calibrated. For the methodological details and theory of modelization in science (see Potochnik et al., 2024). To demonstrate the robustness of the model—that is, the fact that the results do not depend on the specific numerical values chosen—we also carried out a sensitivity analysis. This is a one-at-a-time sensitivity analysis: one parameter is varied at a time, while all the others remain fixed. Each parameter is tested over seven values spanning roughly ±50% around its nominal value, with *N*_MC_ = 5 replicates per condition. We then examine how often each of the six predictions remains valid. The sensitivity analysis therefore does not demonstrate that the parameters are true. It demonstrates something different: the main predictions are not merely an artifact of one specific numerical choice.

4 In active inference, *C* encodes prior preferences as log-preferences rather than as probabilities directly. A log-preference is a real-valued score that specifies how desirable or aversive an outcome is before these scores are transformed into a preferred outcome distribution, typically via a softmax function, *P*^*^(*o*) = softmax(*C*). Thus, higher values of *C*_*i*_ indicate that outcome *i* is more preferred, whereas lower values indicate that it is less preferred or more aversive. In the present model, *C*_low_(*t, c*) denotes the time- and chronotype-dependent log-preference assigned to the low-performance outcome; increasing *C*_low_ makes low performance more tolerable, whereas decreasing it makes low performance more costly in the expected free energy calculation.
