# Supplementary material for: How light reshapes the mind. An active inference framework for the cognitive and emotional effects of indoor lighting
Source: Front Comput Neurosci. 2026 Aug 26;20:1865557. doi: 10.3389/fncom.2026.1865557 (PMC13553800; doi:10.3389/fncom.2026.1865557)
Supplement: Supplementary file 1 [file Supplementary_file_1.pdf]

A Appendix. Symbols Used in this Paper

This appendix collects the symbols used throughout the paper (see Table 10). Where a symbol takes a fixed value in the proof-of-concept simulations, that value is given; symbols whose value varies with scenario, position, chronotype, or time are marked accordingly. Section references point to the place where each quantity is introduced.

Table 10: Symbols used in the model, grouped by role.

Symbol	Meaning	Value / range	Ref.
**Discrete spaces and indices**			
$s_t,\ s_t^*$	Hidden cognitive state / true cognitive state	{focused, distracted, fatigued}	§3.4
N_s	Number of hidden states	3	§3.4
o_t^{perf}	Performance observation	{high, mid, low}_perf	§3.4
o_t^{eye}	Eye-tracking observation	{smooth, effortful, degraded}_scan	§3.4
$N_o,\ N_e$	Number of performance / eye observations	3, 3	§3.4
u_t	Action	{continue, reread, pause}	§3.4
N_u	Number of actions	3	§3.4
$\pi = (u_0, u_1, u_2)$	Policy (action sequence)	$\in U^3$	§3.4
N_π	Number of policies	27	§3.4
t	Decision step	$0, \ldots, T-1$	§3.3
T	Number of decision steps	15	§3.3
Δt	Step duration	20 min	§3.3
c	Chronotype	{lark, intermediate, owl}	§3.3
H	Planning horizon (rollout depth)	3	§3.5.5
**Generative model: likelihood (epistemic channel)**			
A^{perf}	Performance likelihood $P(o^{\text{perf}} \mid s)$	column-stochastic 3×3	§3.5.1
A^{eye}	Eye-tracking likelihood $P(o^{\text{eye}} \mid s)$	column-stochastic 3×3	§3.5.1
A_m^{flat}	Uninformative base likelihood ($m \in \{\text{perf}, \text{eye}\}$)	uniform columns	§3.5.1
A_m^{sharp}	Informative base likelihood	specified, Eq. (9)	§3.5.1
$\phi_{\text{text}}(\ell)$	Text-legibility precision (symmetric Gaussian)	$[0, 1]$	§3.5.1, Eq. (6)
$\phi_{\text{eye}}(\ell)$	Oculomotor precision (asymmetric)	$[0, 1]$	§3.5.1, Eq. (7)

continued on next page

(Table 10, continued)

Symbol	Meaning	Value / range	Ref.
ℓ	Local illuminance at the agent's position	lux	§3.5.1
ℓ^*	Optimal illuminance (precision peak)	500 lux	§3.5.1, Eq. (6)
τ_ℓ	Width of text-legibility precision	400 lux	§3.5.1, Eq. (6)
τ_{eye}	Width of oculomotor precision (glare side)	300 lux	§3.5.1, Eq. (7)
ϕ_{floor}	Baseline (plateau) oculomotor precision	0.40	§3.5.1, Eq. (7)
**Hierarchical extension (§3.2)**			
g	Second-level hidden state (glare level)	{dim, optimal, glare}	§3.2
g^*	True glare level fixed by scenario/position	$\in \mathcal{G}$	§3.2
o^g	Photic (brightness/glare) observation	—	§3.2
$A^g,\ D^g$	Second-level likelihood / prior	column-stochastic	§3.2
$\bar{\phi}_m$	Belief-averaged likelihood precision	$\mathbb{E}_{Q(g)}[\phi_m]$	§3.2
**Generative model: transition dynamics**			
$B^{(u)}(t)$	Transition tensor $P(s_{t+1} \mid s_t, u)$	column-stochastic 3×3	§3.5.2
$B^{(u)}_{\text{fresh}}$	Fresh (early-session) transition dynamics	specified	§3.5.2, Eq. (11)
$B^{(u)}_{\text{tired}}$	Tired (late-session) transition dynamics	specified	§3.5.2, Eq. (11)
$f(t)$	Fatigue factor (1 = fresh, 0 = tired)	$[0, 1]$	§3.5.2, Eq. (12)
t_{onset}	Fatigue onset time	45 min	§3.5.2, Eq. (12)
k	Fatigue sigmoid steepness	6.0	§3.5.2, Eq. (12)
$\delta_{\text{under}}(a)$	Under-arousal drift (focus → distraction)	$[0, 0.35]$	§3.5.2, Eq. (13)
$\delta_{\text{over}}(a)$	Over-arousal drift (focus → fatigue)	$[0, 0.35]$	§3.5.2, Eq. (14)
$a,\ a_{\text{base}}$	Scenario-induced arousal level	≈ 0.32/0.78/1.00 (S1/S2/S3)	§3.3, §3.5.2
**Affective channel (preferences)**			
$C(t, c)$	Log-preference vector over performance obs.	$\in \mathbb{R}^{N_o}$	§3.5.3
$C^{\text{base}}_{\text{low}}(c)$	Baseline log-preference for low_perf	specified	§3.5.3, Eq. (15)

continued on next page

(Table 10, continued)

Symbol	Meaning	Value / range	Ref.
$C_{\text{low}}(t,c)$	Time/chronotype-modulated low_perf preference	Eq. (15)	§3.5.3
$m(t,c)$	Arousal mismatch $a_{\text{base}} - a^*(t,c)$	signed	§3.5.3
κ_c	Arousal-tolerance factor	$-3.0/0.0/+3.5$ (lark/int/owl)	§3.5.3, Eq. (16)
$a^*(t,c)$	Chronotype-specific circadian arousal optimum	≈ 0.63–0.80	§3.5.5, Fig. 9
$P^*(o)$	Preferred outcome distribution softmax(C)	simplex	§3.5.3
**Normative channel and priors**			
D	Initial prior over hidden states	$(0.70, 0.20, 0.10)^\top$	§3.5.4
E_π	Policy prior (normative channel)	per chromaticity context, Eq. (17)	§3.5.4
σ	Chromaticity (hue) context	{warm, neutral, blue}	§3.5.4
$p_{\text{act}}(\sigma)$	Action-tendency distribution by chromaticity	Eq. (17)	§3.5.4
$\mu(\sigma)$	Spectral/chromaticity coefficient (shared driver)	—	§7 (O2)
**Expected free energy and action selection**			
$G(\pi)$	Expected free energy of policy π	nat, Eq. (18)	§3.5.5
$Q(s_t)$	Posterior belief over hidden state	simplex	§3.5.5
$Q(\pi)$	Posterior belief over policies	simplex, Eq. (19)	§3.5.5
Ambiguity(τ)	Expected ambiguity (both modalities)	nat, Eq. (10)	§3.5.1
$H[\cdot]$	Entropy	nat	§3.5.1
$D_{\text{KL}}(\cdot\\|\cdot)$	Kullback–Leibler divergence (risk term)	nat	§3.5.5
$\gamma(t,c)$	Policy precision	Eq. (19)	§3.5.5
γ_0	Baseline policy precision	2.0	§3.5.5, App. C
γ_1	Peak policy precision gain	6.0	§3.5.5, App. C
τ_a	Width of arousal-matching in γ	—	§3.5.5, Eq. (19)
**Behavioural output**			
wpm(t)	Realised reading speed	words/min, Eq. (20)	§3.5.5
$r(s_t^*)$	State-dependent baseline reading rate	$(260, 150, 50)$ wpm	§3.5.5

continued on next page

(Table 10, continued)

Symbol	Meaning	Value / range	Ref.
$\rho(u_t)$	Action productivity multiplier	$(1.0, 0.5, 0.0)$	§3.4
ℓ_{factor}	Mechanical legibility factor	$0.65 + 0.35\,\phi_{\text{text}} \in [0.65, 1]$	§3.5.1, Eq. (20)
**Simulation and experimental design**			
S1, S2, S3	Lighting scenarios (warm-dim / moderate / bright-cool)	—	§3.3
N_{MC}	Number of Monte Carlo replicates	20 (main); 5 (sensitivity)	§3.3, App. C
N_{pts}	Sweep points per parameter (sensitivity)	7	App. C
$\bar{R}$	Mean session risk (per scenario/chronotype)	nat	§5.2
P1–P6	Falsifiable predictions / structural invariants	—	§5.1

B Appendix. Literature Review Analysis

This literature review[5] was conducted from November 2025 to February 2026. We examined studies published from 2000 up to January 2026 on the relationship between lighting and cognitive and emotional functions in many different scenarios and activities, but with special attention to reading. The research was conducted using Google Scholar, PubMed, Scopus, and Web of Science, with combinations of keywords related to lighting (e.g., "lighting," "illumination," "brightness," "color temperature") and cognitive performance (e.g., "cognitive performance," "learning," "memory," "reading"), adapted to the syntax of each database.

The screening process was carried out in two stages: (1) assessment of titles and abstracts, and (2) full-text reading of potentially relevant articles. The initial search yielded approximately 150 records across the four databases. We included peer-reviewed empirical studies that investigated the effects of lighting on at least one cognitive (memory, learning, or attention) or emotional (anxiety, comfort) outcome in human participants. We excluded review articles, publications not in English, and studies lacking explicit measures. Duplicates were identified and removed using an automated comparison of title, authors, and year of publication, followed by a manual check to resolve any ambiguous cases. When multiple versions of the same study were found (e.g., a conference paper and an extended journal article), the most complete and up-to-date version was retained. We also compared these selections with existing literature reviews that focus on more specific aspects (Kızıltunalı, 2023; Chauca et al., 2024).

After screening titles and abstracts, followed by full-text review of potentially relevant articles, 25 studies were selected. For each of these, we analyzed the experimental design and main findings, classifying the results into three categories: (a) effects primarily related to illuminance level (lux, i.e., the amount of luminous flux incident on a surface, equal to one lumen per square meter), (b)

[5]I especially thank Alessio Martino, who made a fundamental contribution to this section.

effects primarily associated with color temperature (Kelvin, i.e., the unit used to express numerically the color temperature, or CCT, of a light source), and (c) effects of the combination of illuminance and color temperature. However, the literature review also highlighted the importance of another aspect: the spectral composition of a light source, i.e., the actual distribution of radiant power across wavelengths; color is a perceptual consequence of this physical distribution.

A formal meta-analysis was not conducted; however, for each of the 25 included studies we qualitatively evaluated several indicators of methodological quality:

1. sample size ($n < 15$, 20–50, > 50 participants);
2. method of assignment to conditions (randomized vs. nonrandomized);
3. control of potential confounding variables (e.g., time of day, exposure to natural light, chronotype, screen use);
4. use of validated cognitive measures (standardized tests vs. ad hoc tasks);
5. duration of exposure to the lighting condition.

Overall, most studies featured small sample sizes and short exposure durations, factors that may limit the generalizability of their findings. In addition, systematic control of circadian and environmental confounders was often incomplete, and only a subset of studies employed fully standardized cognitive instruments.

Table 11 provides a detailed overview of the studies included in the literature review, summarizing key characteristics such as study design, sample, experimental conditions, and main findings. It is intended to complement the conceptual synthesis presented in the main text and to ensure transparency and completeness of the review. The papers are presented in chronological order.

Table 11: Summary of reviewed studies on the cognitive and emotional effects of illuminance (lux), correlated colour temperature (Kelvin), and their combined interactions.

Paper	Study design	Cognitive effects of illuminance (lux)	Cognitive effects of CCT (Correlated Colour Temperature, Kelvin)	Combined effects of K-lux
Knez (2001)	108 paid 18-year-old students (equal gender) participated in a laboratory study in a neutral, temperature-controlled room (21 °C) under three lighting conditions (3000, 4000, 5500 K). They completed mood recognition, room evaluation, problem-solving, and short- and long-term memory tasks following extended reading.	—	The variation of Kelvin did not have significant effects on the emotional level. On a cognitive level, short-term recall was best under warm light (3000 K). Long-term recall showed gender differences: women performed best under daylight (5500 K), while men performed better under warm (3000 K) and cool (4000 K) light. Problem-solving performance was highest under warm light (3000 K), suggesting benefits for tasks requiring selective attention.	—

(Continued on next page)

(Continued from previous page)

**Paper**	**Study design**	**Cognitive effects of illuminance (lux)**	**Cognitive effects of CCT (Correlated Colour Temperature, Kelvin)**	**Combined effects of K-lux**
Naylor & Firth (2008)	Experimental, within-subject repeated-measures design with 20 normally sighted university students (mean age ≈21). Participants completed the Wilkins Rate of Reading Test (WRRT) under four conditions: natural light (≈1200 lux) vs. artificial fluorescent light (≈450 lux) × text on white vs. pink paper.	Participants read significantly faster under artificial lux lighting than under natural lux lighting (mean increase 4.62 wpm; ≈2.7% faster). Reading rate was 177.1 wpm at 450 lux vs. 172.5 wpm at 1200 lux, suggesting that very high illuminance may be slightly detrimental to reading speed, possibly due to glare or excessive page luminance.	CCT was not explicitly manipulated or reported; the contrast is primarily between daylight (1200 lux) and fluorescent artificial light (450 lux).	—
Chellappa et al. (2011)	16 healthy young men (≈24 years). Within-subject, balanced cross-over laboratory study (3 sessions, 1 week apart). Evening/night protocol. Lighting conditions: 6500 K (blue-enriched CFL), 2500 K (warm CFL), 3000 K (incandescent). 2-hour light exposure after dark adaptation. Measures: salivary melatonin, sleepiness, well-being, visual comfort, mental effort; PVT, GO/NOGO, PVSAT.	Illuminance was kept constant (∼40 lux) across all conditions. The study shows that even very low light levels can strongly affect alertness, melatonin, and cognitive performance if spectral composition is blue-enriched.	6500 K produced much stronger melatonin suppression, less sleepiness, higher well-being, better visual comfort, and faster reaction times compared to 2500 K and 3000 K. No significant effects on executive function.	Because lux was fixed, the paper does not test a lux × Kelvin interaction. However, it demonstrates that spectral composition can dominate over intensity: low lux + high CCT (blue-enriched) can be cognitively and biologically very powerful.

(Continued on next page)

(Continued from previous page)

Paper	Study design	Cognitive effects of illuminance (lux)	Cognitive effects of CCT (Correlated Colour Temperature, Kelvin)	Combined effects of K-lux
Barkmann et al. (2012)	Quasi-experimental field study in two schools over nine months. Variable lighting (Philips SchoolVision) vs. standard lighting controls. Lighting scenarios: Standard (300 lux, 4000 K), Concentrate (1060 lux, 5800 K), plus Activate, Relax, etc. Standardised concentration test (d2), reading speed/comprehension, and questionnaires.	—	—	Under the Concentrate program (1060 lux, 5800 K), students made 20.8% fewer omission errors on the d2 test and read 16.8% more words compared to Standard lighting. Reading comprehension showed a positive trend but did not reach significance. High illuminance combined with cooler CCT substantially boosted attention and reading speed.
Mott et al. (2012)	Quasi-experimental study with four third-grade classrooms across an entire school year. Conditions: Focus (1000 lux, 6500 K) vs. Normal (500 lux, 3500 K). Oral reading fluency (ORF) measured at beginning, mid-year, and end-year; motivation and concentration assessed via questionnaires and d2 test.	—	—	A significant lighting × time interaction showed greater ORF gains under 1000 lux/6500 K, maintained across the year. No effects emerged for concentration or motivation, indicating benefits specific to reading fluency.

(Continued on next page)

(Continued from previous page)

Paper	Study design	Cognitive effects of illuminance (lux)	Cognitive effects of CCT (Correlated Colour Temperature, Kelvin)	Combined effects of K-lux
Park et al. (2013a)	32 healthy adults (16 female, mean age ≈26.8). Within-subject, repeated-measures. Task: Raven's Progressive Matrices. Four conditions: WL (2766 K, 300 lux), CL (5918 K, 300 lux), WH (2766 K, 600 lux), CH (5918 K, 600 lux). Frontal and occipital EEG alpha power; subjective valence and arousal ratings.	Higher brightness caused significantly higher valence (people felt happier). Occipital alpha power was lower in high light than low light, suggesting brightness affects visual cortex activation. Brightness modulates emotional state and visual cortical activity, but does not show a simple "more light = better cognition" effect.	Warm light (low K) made subjects feel more relaxed (lower arousal). Colour temperature did not significantly affect valence, only arousal. No strong main effect of CCT alone on alpha power.	Highest alpha power (better focused internal processing) occurred in congruent combinations: warm + low light (WL) and cool + high light (CH). Lower alpha power in mismatched conditions (WH, CL). Cognitive brain state is determined by the combination of brightness and CCT, not by either alone.
Park et al. (2013b)	22 adults (11 female, mean age ≈23). Conditions: 3000 K vs. 7100 K; 150 lux vs. 700 lux (cool–dark, cool–bright, warm–dark, warm–bright). Sternberg working memory task (set sizes 3, 5, 7). EEG: frontal theta power; occipital ERPs (P1/N1).	Higher illuminance (700 lux) reduced frontal theta power during working memory retention (interpreted as a change in mental effort) and increased N1 amplitude. Brightness altered early attentional/sensory processing but did not translate into better or worse task performance.	CCT had minimal/no significant effects across main outcomes. Key EEG effects were attributed primarily to illuminance.	Lighting effects are largely explained by lux; no strong evidence of a meaningful lux × Kelvin interaction on behavioural performance. Mood/emotion changes were not measured.

(Continued on next page)

(Continued from previous page)

Paper	Study design	Cognitive effects of illuminance (lux)	Cognitive effects of CCT (Correlated Colour Temperature, Kelvin)	Combined effects of K-lux
Keis et al. (2014)	58 high-school students from two schools split into blue-enriched vs. normal lighting groups. Four conditions combining normal and blue-enriched light during lessons and tests (normal/normal, normal/blue, blue/normal, blue/blue).	—	—	Under 5500 K and 300 lux lighting, cognitive performance increased. Full blue-enriched light exposure improved processing speed, concentration, and visual memory; students reported greater appreciation for this condition.
Huiberts et al. (2015)	64 students (32 male, 32 female; mean age 21.4). Two counterbalanced lab sessions in an office-like setting. Two illumination levels (200 and 1000 lux) tested on digit span and n-back tasks. Participants completed four 15-min blocks after a 100-lux baseline.	Higher light (1000 lux) improved speed and accuracy on easy tasks; lower light (200 lux) led to more stable performance on difficult tasks. More light does not necessarily improve performance; illumination should be matched to task demands.	Held constant at 4000 K — no effects.	—
Choi & Suk (2016)	Series of lab + field experiments in elementary-school classrooms using tunable LEDs at three presets: 3500 K (“Relax”), 5000 K (“Standard”), 6500 K (“Energy”), all at ∼500–600 lux at desk level. Pre-test–post-test control-group design plus lab tests.	Illuminance was intentionally kept in a narrow band (∼450–570 lux); the study effectively isolates CCT effects.	6500 K produced the greatest number of correct answers in arithmetic tests. 3500 K was associated with higher comfort and recommended for rest/relaxation. 5000 K suggested as default for reading and typical classroom activities.	Within ≈500 lux, CCT clearly differentiated functions: 3500 K for rest; 5000 K for ordinary reading/lecture; 6500 K for intensive cognitive work with improved performance. Because illuminance was held roughly constant, the main conclusion is about CCT.

(Continued on next page)

(Continued from previous page)

Paper	Study design	Cognitive effects of illuminance (lux)	Cognitive effects of CCT (Correlated Colour Temperature, Kelvin)	Combined effects of K-lux
Hartstein et al. (2018)	38 healthy preschool children (4.5–5.5 years; 21 female). Windowless lab; tasks on a laptop. Tunable LED ceiling luminaires. Participants assigned to experimental (3500 to 5000 K) or control (constant 3500 K) groups. Go/No-Go and Hearts & Flowers tasks assessed attention, inhibition, and cognitive flexibility.	Illuminance was held approximately constant.	Because lux was controlled, the study mainly isolates CCT effects. Go/No-Go: no lighting effect on accuracy; reaction time improved similarly in both groups (practice effect). Hearts & Flowers (task switching): higher CCT (5000 K) produced greater improvement in switch-trial accuracy than remaining at 3500 K.	With brightness held constant, changing CCT from 3500 to 5000 K was enough to improve task-switching accuracy on switch trials.
Ram & Bhardwaj (2018)	40 young adults (∼20 years; 20 female). Four light sources at controlled 400 lux: CFL (∼3400 K), fluorescent tube (∼3000 K), tungsten incandescent, and LED. Reading rate, contrast sensitivity, colour vision, and subjective comfort assessed after each condition.	All conditions used 400 lux; this paper does not test different lux levels — effects are purely spectral/source-type at constant illuminance.	Because the CCT range is narrow (≈3000–3400 K) and multiple lamp characteristics change simultaneously (spectrum shape, flicker, CRI), effects on reading appear driven more by light-source quality/spectrum than by CCT as an abstract parameter.	—

(Continued on next page)

(Continued from previous page)

Paper	Study design	Cognitive effects of illuminance (lux)	Cognitive effects of CCT (Correlated Colour Temperature, Kelvin)	Combined effects of K-lux
Zhu et al. (2019)	Laboratory, 2 (illuminance: 200 vs. 1200 lux) × 2 (CCT: 3000 vs. 6500 K) × 2 (time of day: morning vs. afternoon) mixed design with 60 healthy young adults (30 female). Tasks: Go/No-Go, 2-back, facial expression recognition, long-term word recognition; mood (PANAS) and alertness ratings.	Bright light (1200 lux) made participants less sleepy and more positively toned than dim light (200 lux). For Go/No-Go and 2-back, reaction times were slowest in the low-illuminance warm condition, and bright light improved performance.	Go/No-Go accuracy showed a main effect of CCT: participants were less accurate in cool (6500 K) than warm (3000 K) light. For 2-back, CCT mainly entered through interactions with illuminance.	The worst cognitive performance consistently appeared in low-illuminance + warm light (200 lux, 3000 K). Best long-term word recognition occurred in high-illuminance cool conditions (1200 lux, 6500 K) for neutral words in the afternoon. Bright light is reliably beneficial for alertness, mood, inhibition, and working memory; CCT modulates these effects.
Grant et al. (2021)	Randomized, within-subjects trial with sleep-restricted college-aged adults exposed to four polychromatic white LED spectra differing in short-wavelength enrichment (melanopic EDI) at roughly similar photopic illuminance. Cognitive battery: working memory/processing speed, motor sequence learning, vigilance (PVT), declarative memory; subjective alertness.	Lux was held constant.	Higher melanopic EDI (≈ higher CCT) produced lower subjective sleepiness and better performance on some cognitive measures than low-melanopic (≈ lower CCT) conditions.	—

(Continued on next page)

(Continued from previous page)

**Paper**	**Study design**	**Cognitive effects of illuminance (lux)**	**Cognitive effects of CCT (Correlated Colour Temperature, Kelvin)**	**Combined effects of K-lux**
Ru et al. (2021)	Within-subjects lab study with healthy adults tested on two separate days under low (∼100 lux) vs. high (∼1000 lux), both at ≈6500 K. Tasks: sustained attention (PVT), response inhibition (Go/No-go), conflict monitoring (Flanker), working memory (2-Back, PVSAT); repeated measures of sleepiness, mood, and light appraisals.	High vs. low illuminance did not significantly affect PVT or Flanker. For Go/No-go, high illuminance sped responses only for the easy version. For working memory, 1000 lux improved accuracy and speed on 2-Back and accuracy on PVSAT; benefits were larger for easier trials.	CCT was held constant (6500 K); this study cannot speak to Kelvin effects directly.	In a bright-cool office (6500 K), raising illuminance from 100 to ∼1000 lux selectively benefits working-memory and simple response-inhibition performance, especially for less difficult task versions. No uniform "bright is always better" effect; intellectual/working-memory tasks can profit from higher lux.
Shishegar & Boubekri (2022)	Field, crossover intervention with 21 healthy older adults (mean age ≈77). Two 9-day lighting interventions separated by a 2-week washout. Whole-day dynamic scheme: blue-enriched morning light decreasing to warm, dim evening light. Cognitive performance (TMT, DSST) and mood (GDS, PANAS) assessed.	Increasing daytime illuminance to 200–500 lux (from chronically dim) produced significant improvements in mood and cognitive performance (faster TMT, better DSST). Moving to appropriately bright daytime lighting benefits older adults' attention, processing speed, and global cognition.	Adding spectrum tuning (high CCT morning → neutral afternoon → low CCT evening) produced significantly larger improvements in both mood and cognitive measures than illuminance changes alone.	High lux + high CCT in the morning supports better daytime cognitive functioning and mood. Variable illuminance alone was beneficial, but variable illuminance + variable CCT was better, indicating that Kelvin modulates the cognitive benefits of lux across the day.

(Continued on next page)

(Continued from previous page)

Paper	Study design	Cognitive effects of illuminance (lux)	Cognitive effects of CCT (Correlated Colour Temperature, Kelvin)	Combined effects of K-lux
Castilla et al. (2023)	Immersive VR replica of a university classroom; geometry, materials, and CCT held constant. Only illuminance manipulated at 100, 300, and 500 lux on the desk plane. 142 university students completed memory tasks while HRV, EEG, and EDA were recorded.	Memory performance was highest at 100 lux, with significant declines at 300 and 500 lux (which did not differ from each other). Neurophysiological markers showed higher activation at 100 lux, mirroring better memory; higher illuminance was associated with reduced arousal/engagement.	CCT was held fixed.	—
Chauca et al. (2023)	Experimental + review-based study on classroom LED lighting combining technical characterisation and student performance questionnaires to identify optimal lux and CCT. Integrates and interprets results from several prior empirical case studies.	≈400 lux associated with optimal long-term memory. Near-linear increase in attention with illuminance, reaching notably higher attention at ∼1060 lux compared with 300 lux.	6500 K is repeatedly associated with higher learning, especially for arithmetic problem solving.	6500 K combined with 500–600 lux yields more correct answers in arithmetic; ≈400 lux appears optimal for long-term memory; ∼1000–1060 lux for peak attention. The paper argues for differentiated settings: moderate lux for memory, higher lux for attention, cool CCT for intensive learning.

(Continued on next page)

(Continued from previous page)

Paper	Study design	Cognitive effects of illuminance (lux)	Cognitive effects of CCT (Correlated Colour Temperature, Kelvin)	Combined effects of K-lux
Nole Fajardo et al. (2023)	Immersive VR study comparing four classroom configurations differing in lighting, wall colour, and geometry. Lighting manipulations involved different levels of illuminance and CCT within realistic classroom ranges. University students performed attention and memory tasks; preferences collected with analyses segmented by gender.	—	—	Rather than a monotonic rule, the study shows that changing illuminance and CCT meaningfully modulates attention and memory, with gender-specific sensitivities. The work strongly supports prioritising lighting (lux + Kelvin) over other spatial features for cognitive optimisation.
Zhou & Pan (2023)	Full-scale mock classroom, 3 illuminance levels (300, 500, 750 lux) × 3 CCTs (2700, 4000, 6500 K) × 3 reading durations (15, 30, 60 min). Twelve young adults performed a paper-based number-correction task simulating reading while EEG was recorded.	—	—	Higher illuminance and higher CCT improved reading efficiency and reduced EEG-defined fatigue, but the optimal combination depended on reading duration: (a) 15 min: best at 500 lux & 6500 K; (b) 30 min: best at 500 lux & 4000 K; (c) 60 min: best at 750 lux & 6500 K. Lux and Kelvin interact with task duration; there is no single static optimal setting.

(Continued on next page)

(Continued from previous page)

Paper	Study design	Cognitive effects of illuminance (lux)	Cognitive effects of CCT (Correlated Colour Temperature, Kelvin)	Combined effects of K-lux
Mostafavi et al. (2024)	VR-based office simulation with five lighting conditions: 2000 K (Warm-Dark, Warm-Bright), 4600 K (Neutral-Moderate), 7200 K (Cold-Dark, Cold-Bright); illuminance ≈100, 400, 700 lux at workplane. Tasks: working memory (BDST) and visual memory (VMT) during morning, afternoon, and evening sessions.	Brighter conditions (∼700 lux) generally yielded better scores on some memory tasks (especially VMT) than dark (∼100 lux). Effects were task- and time-of-day-dependent; benefits of higher illuminance were clearer for VMT and at specific times.	Very warm light (2000 K) tended to be disadvantageous for demanding working-memory performance, particularly in the afternoon. Neutral 4600 K frequently produced stable, relatively good performance with moderate brightness (400 lux).	No single optimal lux–Kelvin pair. Broad pattern: bright and neutral/cool conditions (≈400–700 lux, ≥4600 K) supported working and visual memory better than dim, very warm light (≈100 lux, 2000 K), especially later in the day. Illuminance has a somewhat stronger and more consistent influence than CCT; CCT modulates the effect.
Jung et al. (2024)	Laboratory study in an artificial climate chamber with 16 healthy adults in their 20s. Nine office-type LED conditions: 3 CCTs (4000, 5000, 6500 K) × 3 illuminances (200, 500, 800 lux). Cognitive battery (primary and complex functions), psychophysiological measures, and subjective workload and fatigue; mixed linear models and regression.	Within the realistic office range (200–800 lux), higher illuminance was associated with better primary cognitive performance, especially attention.	Higher CCT (toward 6500 K) significantly improved memory and some executive-function measures when illuminance was held constant.	Lux and Kelvin reliably enhance lower-level cognitive processes (attention, basic memory) within normal office ranges, but do not guarantee improvements in higher-order cognition or psychophysiological indicators.

(Continued on next page)

(Continued from previous page)

Paper	Study design	Cognitive effects of illuminance (lux)	Cognitive effects of CCT (Correlated Colour Temperature, Kelvin)	Combined effects of K-lux
Li & Yao (2025)	Within-subject 3 × 3 design: 3 illuminance levels (200, 500, 1000 lux) × 3 CCTs (2730, 3930, 5870 K) with tunable LED ceiling luminaires in a simulated office. Healthy young adults read texts of two difficulty levels on a monitor; eye tracker recorded gaze metrics; Score of Reading Efficiency (SRE) computed; brightness, visual comfort, and pupil size measured.	SRE increased monotonically from 200 → 500 → 1000 lux in both easy and difficult texts; significantly better reading efficiency at 1000 vs. 200 lux and typically at 500 vs. 200 lux.	CCT alone did not significantly change SRE or comfort in main-effect analyses, although comfort votes tended to rise with higher CCT (more participants rating “comfortable” at 5870 K than at 2730 K).	Lux and CCT interacted: the positive effect of higher illuminance on SRE became more pronounced at higher CCTs (1000 lux at 5870 K yielded the highest reading efficiency). Higher illuminance and higher CCT produced smaller pupil sizes, negatively correlated with SRE, suggesting that bright, relatively cool light supports more efficient visual–cognitive processing during reading.
Mott et al. (2025)	High-school field experiment in four classrooms (students randomly assigned by grade-level strata). Blinds closed; illuminance and CCT carefully measured at desk and eye level. Energy (intervention): ≈4120 K, ∼436 lux, with higher spectral concentration in the blue-to-cyan band (440–520 nm). Control: ≈3789 K, ∼486 lux, normal blue content. Students completed a practice ACT Reading Comprehension test under assigned lighting.	—	—	With illuminance near-matched (∼500 lux) and CCT in the cool-but-not-extreme range (∼3800–4200 K), Energy-classroom students scored significantly higher on the practice ACT reading test (adjusted means ≈53.2 vs. 45.5). The improvement is attributed mainly to spectral composition (more blue-to-cyan) interacting with moderate lux and cool CCT to enhance alertness and reading comprehension.

(Continued on next page)

(Continued from previous page)

**Paper**	**Study design**	**Cognitive effects of illuminance (lux)**	**Cognitive effects of CCT (Correlated Colour Temperature, Kelvin)**	**Combined effects of K-lux**
Zhu et al. (2025)	Within-subjects lab experiment with 12 young adults reading on an e-book under two CCTs (4000 and 6500 K) and two melanopic EDI levels per CCT, at ∼100 lux vertical / 300 lux horizontal. Reaction-time task and memory game while subjective (comfort, sleepiness, fatigue) and physiological measures were recorded.	Illuminance on the desk held at ∼300 lux; the study does not test different lux levels. Effects are driven by spectral changes at a fixed illuminance.	At the same illuminance, 4000 K produced more sensitivity to changes in melanopic EDI than 6500 K: more physiological parameters and general fatigue ratings differed between low vs. high mel-EDI at 4000 K, while effects at 6500 K were smaller.	Cognitive performance (reaction times and memory-game performance) did not show robust, systematic differences across mel-EDI or CCT conditions at 300 lux; lighting mainly modulated alertness and comfort rather than learning/memory outcomes in this e-reading context.

C Sensitivity Analysis

A one-at-a-time (OAT) sensitivity analysis tests whether the six predictions hold across plausible variations in eight key parameters. Each parameter is swept across $N_{\text{pts}} = 7$ evenly spaced values spanning approximately ±50% of its nominal value (Table 12), with all other parameters held fixed and $N_{\text{MC}} = 5$ replicates per condition. The metric for each cell is the mean pass rate across the seven sweep values. Figure 10 summarises the mean pass rate for each prediction across the sweep, and Figure 11 resolves the same result along each parameter axis, showing where each prediction degrades and how sharply.

Table 12: Parameters swept, with nominal values and ranges.

Parameter	Symbol	Nominal	Range	Channel
Legibility width	τ_ℓ	400 lux	200–700	Epistemic
Peak precision	γ_1	6.0	2–12	Affective
Baseline precision	γ_0	2.0	1–4	Affective
Fatigue onset	t_{onset}	45 min	15–90	Temporal
Fatigue rate	k	6.0	3–12	Temporal
Lark tolerance	κ_{lark}	−3.0	−6–−1	Affective
Owl tolerance	κ_{owl}	+3.5	+1–+7	Affective
Over-arousal threshold	a^*_{over}	0.78	0.60–0.95	Affective

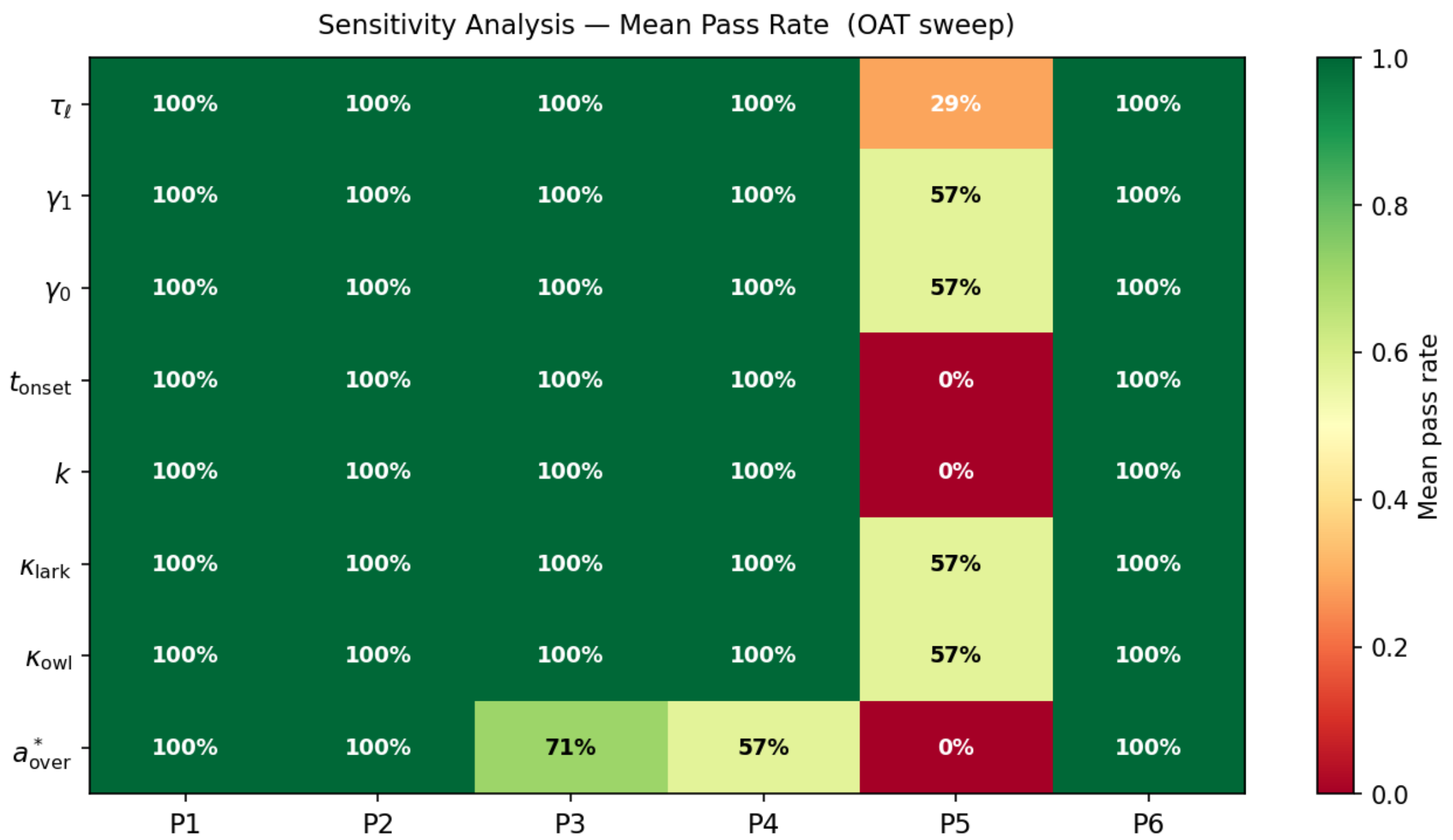

Figure 10: Mean pass rate across the parameter sweep for each prediction. Green ≥ 80%; red ≤ 40%.

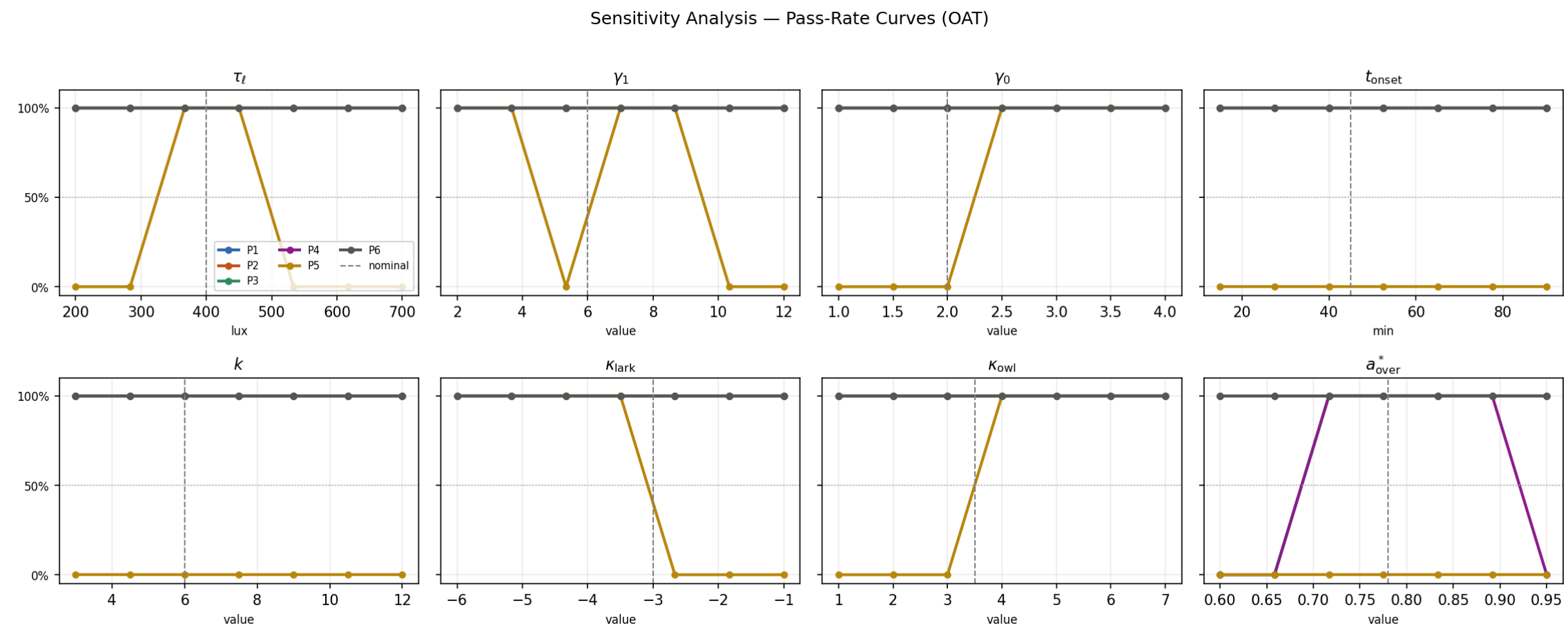

Figure 11: Pass-rate curves along each parameter sweep. Dashed vertical lines mark the nominal value. Flat curves indicate structural robustness; steep drops indicate parameter sensitivity.

Table 13 reports the mean pass rate for each parameter-prediction combination.

Table 13: Mean pass rate (%) across the sweep. Bold $\geq 80\%$; italic $\leq 40\%$.

Parameter	P1	P2	P3	P4	P5	P6
τ_ℓ	**100**	**100**	**100**	**100**	*29*	**100**
γ_1	**100**	**100**	**100**	**100**	57	**100**
γ_0	**100**	**100**	**100**	**100**	57	**100**
t_{onset}	**100**	**100**	**100**	**100**	*0*	**100**
k	**100**	**100**	**100**	**100**	*0*	**100**
κ_{lark}	**100**	**100**	**100**	**100**	57	**100**
κ_{owl}	**100**	**100**	**100**	**100**	57	**100**
a^*_{over}	**100**	**100**	71	57	*0*	**100**

Three robustness tiers emerge.

Tier 1 — Structural invariants (P1, P2, P6). All three pass at 100% across every sweep. P1 and P2 test properties that follow by construction from the architecture: **A** is chronotype-blind (P1), and κ_{lark} and κ_{owl} have opposite signs (P2). P6 is invariant because $\kappa_{\text{int.}} = 0$ structurally eliminates scenario sensitivity for the intermediate chronotype regardless of all other parameter values.

Tier 2 — Conditionally robust (P3, P4). Both pass at 100% under six of the eight sweeps and degrade only when a^*_{over} is moved above 0.85. In that regime, S2 itself begins to induce mild over-arousal drift in **B**, partially contaminating the legibility gradient that P3 depends on, and reducing the over-arousal penalty at the centre position that P4 depends on. This degradation is interpretable: it occurs precisely when the specific mechanism behind each prediction is attenuated, not when the architecture as a whole is wrong.

Tier 3 — Parameter-sensitive (P5). P5 passes at 0% under three sweeps (t_{onset}, k, a^*_{over}) and at 29–57% under the rest. This prediction requires a precise balance between the owl's tolerance advantage (κ_{owl}), its precision advantage (γ_1), and the temporal structure of the session (fatigue determining how much of the session both channels are informative). Varying any of these

independently tips the balance. P5 should therefore be treated as an exploratory prediction: its confirmation would inform the calibration of the affective and temporal parameters, but its failure in an experiment would not challenge the three-channel architecture.

P1, P2, and P6 are the appropriate targets for a minimal initial empirical study: they would survive arbitrary reparametrisation. P3 and P4 together constitute the critical experiment. P5 is informative about parameter values.
